# AI technology specialization and national competitiveness

**DOI:** 10.1371/journal.pone.0301091

**Published:** 2024-04-04

**Authors:** Youngsam Chun, Jisoo Hur, Junseok Hwang

**Affiliations:** 1 Technology Management, Economics and Policy Program (TEMEP), Seoul National University, Gwanak-gu, Seoul, South Korea; 2 Global R&DB Center, Seoul National University, Gwanak-gu, Seoul, South Korea; 3 Institute of Convergence Technology, Korea Telecom (KT), Seoul, South Korea; 4 International Technology Professional Program (ITPP), Seoul National University, Gwanak-gu, Seoul, South Korea; Roma Tre University: Universita degli Studi Roma Tre, ITALY

## Abstract

This study investigates the factors influencing specialization in artificial intelligence (AI) technology, a critical element of national competitiveness. We utilized a revealed comparative advantage matrix to evaluate technological specialization across countries and employed a three-way fixed-effect panel logit model to examine the relationship between AI specialization and its determinants. The results indicate that the development of AI technology is strongly contingent on a nation’s pre-existing technological capabilities, which significantly affect AI specialization in emerging domains. Additionally, this study reveals that scientific knowledge has a positive impact on technological specialization, highlighting the necessity of integrating scientific advancements with technological sectors. Although complex technologies positively influence AI specialization, their effect is less pronounced than that of scientific knowledge. This suggests that in rapidly advancing fields, such as AI, incorporating new scientific knowledge into related industries may be more advantageous than simply advancing existing technologies to outpace competitors. This insight points nations toward enhancing AI competitiveness in new areas, emphasizing the vital importance of both scientific and technological capabilities, and the integration of novel AI knowledge with established sectors. This research offers critical guidance for policymakers in less technologically and economically developed countries, as these nations may not have the technological infrastructure required to foster AI specialization through increased technical complexity.

## Introduction

In recent years, intensifying global competition to achieve national competitiveness in artificial intelligence (AI) technology has significantly increased interest in technological specialization [1, 2]. Technological specialization is defined as a country’s emphasis on producing and developing specific technologies when that country has a comparative advantage. This competitive environment has resulted in a significant increase in academic research and patent publications on AI. From 1960 to early 2018, approximately 340,000 AI-related patent families and over 1.6 million scientific papers were published, with the number of papers approximately 4.7 times that of patents [3]. This disproportionate growth raises an essential question: Does an increase in scientific publications significantly influence a nation’s technological specialization in AI?

In the current knowledge-driven economy, which is characterized by intense competition among nations for technological supremacy, the development of new technologies through innovative scientific concepts has become increasingly vital. However, national-level exploration of this phenomenon has been limited, with a few significant exceptions, such as Catalan et al.’s study [4]. This research uniquely points out that previous studies have typically focused on technologies or scientific areas in isolation, neglecting to explore their interconnection. Specifically, they investigate whether a nation’s intrinsic scientific capabilities can determine its potential for technological specialization. Traditionally, research has focused on regional aspects of knowledge spillover by examining the transformation of scientific knowledge into technological innovations [5–7]. University research generally fosters innovation within its region, a trend largely due to knowledge spillover confined by geographical boundaries, as Jaffe et al. [8] observed.

This study investigated the intersection of scientific knowledge and technological specialization in AI at the national level. We analyzed data on AI-related scholarly articles and patents from 170 countries over a span of four decades, from 1980 to 2019. This timeframe is particularly significant, as it includes key historical events that have led to recent breakthroughs in the evolution of AI technology. For example, it covers the period following the ’AI winter,’ a phase in the late 1980s and early 1990s characterized by reduced interest and investment in AI that coincided with the market collapse of early AI technologies such as expert systems and machine learning algorithms. The late 1990s witnessed a resurgence of optimism in relation to AI, as evidenced by a surge in related publications, which were often linked to advancements in machine learning and neural networks [9]. The early 2000s saw a significant increase in AI patent filings, reflecting increased commercial attention and major technological breakthroughs, particularly in the realm of deep learning techniques [10], which were pivotal in the development of AI [11].

Our dataset also includes pivotal research and patents related to Google Brain’s Transformer, as introduced by Vaswani et al. [12] in ’Attention Is All You Need.’ This discovery marked a significant breakthrough in natural language processing and laid the groundwork for advanced language models such as ChatGPT. This period was crucial in the evolution of AI, particularly in language models, as evidenced by the explosion of research and technological applications stemming from this model [13, 14]. These developments have profoundly influenced the contemporary AI landscape, underscoring the importance of groundbreaking scientific discoveries in driving technological advancement and specialization.

Through the analysis of a comprehensive dataset, this study aims to shed light on how the expanding corpus of scientific knowledge has impacted the technological specialization of countries in AI-related fields. By considering both the historical and contemporary contexts of AI development, this approach seeks to elucidate the intricate relationship between scientific progress and technological innovation in the rapidly evolving domain of AI. Such an analysis offers a nuanced understanding of the dynamic interplay between these two critical elements, contributing significantly to our understanding of the developmental trajectory of AI.

Our findings indicate that both scientific and technological capabilities contribute significantly to a country’s AI specialization. Notably, scientific knowledge that aligns closely with a country’s existing technological strengths tends to positively affect its technological comparative advantage, implying beneficial knowledge spillover from science to technology when there is a certain level of alignment between the two domains. Furthermore, the influence of scientific capabilities on AI specialization appears to be more pronounced than that of technological complexity, although complex technologies contribute positively to AI specialization.

The insight derived from this study is particularly pertinent to policymakers in developing countries, where technological resources may be more constrained. In these technologically and economically less developed nations, focusing on gaining a comparative advantage in sectors closely linked to the latest AI knowledge presents a more feasible strategy than attempting to emulate the approach of advanced countries, which often involves complicating existing technologies to remain ahead of competitors. This underscores the critical need to integrate advanced scientific knowledge into industries that are strategically important for AI specialization. Adopting this strategy is essential for countries striving to establish or strengthen their niche in the fast-paced AI landscape. In this arena, where both scientific and technological breakthroughs are key to maintaining a competitive edge, a focused approach to leverage scientific advancements can offer significant strategic benefits.

Our study marks a significant advancement by transitioning from a unidimensional approach to a multidimensional perspective and by intertwining scientific and technological knowledge spaces. We utilized the multilayer network approach proposed by Pugliese et al. [15], leveraging co-occurrence data to map out the interactions within the national innovation system more effectively. This method surpasses the boundaries of conventional approaches by accounting for the diverse and concurrent interactions between scientific research and technological economic activities [4, 16]. This comprehensive perspective equips policymakers with holistic insight and facilitates the development of integrated AI strategies that acknowledge the interdependent nature of research and innovation. In a rapidly evolving technological landscape, adopting a strategy that leverages the most current knowledge to extend comparative advantage into new areas—rather than merely complicating existing technologies to deter competitors—has become increasingly vital. The findings of this study provide valuable guidance for policymakers in crafting AI technology policies, underscoring the necessity for a multidimensional approach that addresses both science and technology simultaneously.

## Literature review

### RCA-based technology specialization

Technology specialization refers to a country that focuses on its production and expertise in specific technologies in regard to which it has a comparative advantage. Countries’ economic prosperity is closely linked to their specialization in specific technologies, products, and skills. This principle, dating back to the work of Marshall [17] and Jacobs [18], which was further explored by Glaeser et al. [19], emphasizes the significance of specialization. Balassa [20] introduced the concept of comparative advantage, suggesting that a country excels in producing a specific product when its production surpasses the global average. This concept has led to the development of the Revealed Comparative Advantage (RCA) index, a pivotal economic measure used to assess a country’s competitive edge in various industries or trades. The RCA index calculates the proportion of a country’s exports of a particular good or service relative to its share of global exports. An RCA greater than 1 indicates a comparative advantage, signifying greater proficiency in producing that good or service compared with other countries.

The RCA index, traditionally used to assess product specialization, is also highly applicable to technological specialization. Numerous recent studies have employed RCA to gauge technological competitiveness at various levels, including regional, city, and national [16, 21–24]. Particularly noteworthy is the work of Catalan et al. [4], who extended the RCA concept to measure the relative advantage of technologies across multiple countries engaged in their development. They investigated the influence of a country’s scientific knowledge based on its capacity to develop new technologies and found a positive correlation between a strong scientific knowledge base in a particular domain and a country’s ability to innovate in that domain. This finding underscores the significant role that a country’s scientific infrastructure plays in driving technological advancement.

The process of technological specialization is path-dependent, meaning that existing capabilities determine the trajectory of technology specialization. A country’s transition toward new technologies is heavily influenced by its existing technological and industrial capabilities [25, 26]. Therefore, countries tend to specialize in technologies related to their pre-existing capabilities. This is consistent with the principle of relatedness, which suggests that countries tend to diversify into areas related to their current technological and industrial capabilities [21, 23, 27]. Boschma et al. [28] demonstrated the path-dependent nature of technology evolution, including specialization and decline, in U.S. cities. They expanded RCA’s utility by introducing ENTRY and EXIT metrics using patent class relatedness to predict technological shifts. Their results suggested that regions often develop industries akin to their existing ones. They found that a 10% increase in technological relatedness increases the likelihood of adopting a new technology (ENTRY) by 30% and reduces the obsolescence (EXIT) of current technologies by 8%.

Factors influencing technological specialization extend beyond mere technological relatedness to include technological complexity. The significance of technological complexity in bolstering specialization is substantial. Profound and intricate knowledge in a specific field positively influences the introduction of novel technologies, thereby reinforcing specialization [29, 30]. Additionally, this complexity is often correlated with enhanced economic value owing to its geographic concentration and the difficulties it presents for widespread dissemination, which poses challenges for other countries in terms of replication and imitation [8, 31]. As such, nations tend to exhibit distinct patterns of specialization along their developmental trajectories, gravitating toward more complex and economically valuable technologies to rapidly monopolize economic benefits [22]. The aggregation of complex knowledge in specific countries has led to the emergence of more advanced and sophisticated export patterns. As countries progress, they refine and augment their distinctive technological capabilities [32, 33], emphasizing the pivotal role of technological complexity in shaping and driving the trajectory of technological development.

However, excessive specialization in a particular technology carries the risk of technological lock-in [34]. This occurs when an economy becomes excessively reliant on a specific technology, leading to significant transition costs or barriers to adopting new technologies. The risk of technological lock-in is heightened when the focus is on increasing the complexity of a specific technology, as this can lead to path dependence, with future technological developments heavily influenced by past decisions [35]. This scenario limits flexibility and adaptability, potentially hindering economic progress and innovation [34–36].

Although RCA-based measures of technological specialization are not flawless, they offer considerable benefits. The ENTRY and EXIT indicators derived from RCA do not directly measure entrance or exit from a specific technological field. Rather, they assess the comparative advantage in that domain, thereby adding robustness and minimizing the impact of external volatility [16, 28, 37]. When analyzing patent data to gauge technological specialization, using RCA-based ENTRY indicators is more advantageous than simply counting patents. This approach counters the variability inherent in patent data, which can be influenced by factors such as changes in patent laws, varying patenting behaviors across countries, and technological shifts [38]. By focusing on relative strengths rather than absolute patent counts, the RCA-based specialization measure effectively addresses these variabilities, aligning with economic complexity theory [37].

Despite its limitations, the RCA-based ENTRY metric is invaluable for quantifying technological specialization. Its effectiveness lies in evaluating specialization through relative comparisons among countries rather than relying solely on the absolute number of specific technologies. This comparative approach provides a more resilient and nuanced understanding of the technological strengths and weaknesses of a nation. It yields insight that is less affected by external factors, such as patenting activity fluctuations or global market changes, thus offering a more stable and insightful perspective on technological specialization.

### AI specialization in the science-technology nexus

Exploring AI specialization through the science-technology nexus entails a deep exploration of how scientific research underpins technological progress in AI. This relationship is reciprocal; AI technological breakthroughs often arise from foundational scientific research, and technological advances pave the way for new scientific inquiries. Understanding this interplay offers valuable insight into the dynamics that drive AI specialization, the birth of novel AI technologies, and their sector-wide adoption.

The role of science in fostering new technologies with a comparative edge is crucial in today’s knowledge economy, which is characterized by fierce global competition for technological leadership. However, this crucial aspect has been explored less at the country level despite its significance in the regional dynamics of knowledge spillover and the transformation of scientific knowledge into technological innovations [8]. This spillover, often geographically bound to areas surrounding universities and research institutes, highlights the importance of local knowledge in innovation processes [5–7].

Research suggests that knowledge spillovers in the domain of AI tend to be localized, supporting the need for targeted subnational and national AI development policies [39]. Notable examples include AI powerhouses such as DeepMind in London and the Vector Institute in Toronto, where their leading researchers were based [40]. This phenomenon of AI expertise clustering in certain regions is a global trend, with innovation hubs emerging in cities such as Silicon Valley, Berlin, Seattle, London, Boston, Shanghai, Toronto, and Montreal [40]. Several factors drive this trend, including the rapid pace of scientific advancements in AI. Companies aim to promptly integrate the latest scientific findings into their technologies to shorten the time required for scientific knowledge to be transformed into technological applications [41, 42].

However, the transition from scientific theory to the technological application of AI has not always been swift. Alan Turing’s 1950 concept of a ’thinking machine’ laid the groundwork for AI, but it took decades for this concept to influence tangible technological advancements due to a series of progressions and setbacks marked by the varying prominence of different technologies [43]. The advent of deep learning, particularly following the significant contributions of Hinton et al. in 2012, marked a turning point in the evolution of AI [10, 44]. Currently, AI is recognized as a critical technology across various industries, with machine-learning methods proving particularly transformative [45].

The progression of AI technologies is closely tied to specific scientific breakthroughs. For example, the integration of deep learning into numerous computer science disciplines has accelerated the advancement of AI [39]. The broadening applicability and exponential development of AI are evidenced by the rapid increase in academic publications and patent registrations, which has been particularly noticeable since the early 21st century [11, 39]. This surge is a result of sustained research commitment and continual innovation, which have been maintained even during periods of skepticism [43].

Global rivalry in AI specialization has escalated, with the US and China having an edge owing to their access to substantial datasets. Stringent European data laws may impede the region’s ability to develop sophisticated AI [39, 46]. China, the USA, Canada, and other Asian countries such as Singapore and Korea, have emerged as global AI leaders [39]. International competition has led to an increase in both academic and patent publications related to AI. Between 1960 and early 2018, nearly 340,000 AI-related patent families and over 1.6 million scientific papers were published. The number of papers published is approximately 4.7 times the number of patents [3]. The boom in the development of AI-related scientific papers began approximately 10 years prior to patents, with an average annual growth rate of 8 percent between 1996 and 2001 [3].

While it might seem logical for an increase in scientific and technological knowledge to lead to a nation’s specialization in AI, the situation is more nuanced. For example, AI patenting is predominantly concentrated among a small number of large firms, indicating that simply increasing knowledge does not necessarily lead to widespread AI specialization [47]. Furthermore, the diversity of AI research has stagnated, adding complexity to the relationship between knowledge accumulation and AI specialization [47].

The situation in less technologically and economically developed countries with limited AI-related technical capabilities is particularly challenging. Despite the importance of understanding how these countries specialize in AI, this field has not received sufficient attention. Liu et al. [41] argue that for nations with lower levels of technological development, strategies should be formulated to foster AI development and application, promoting knowledge creation and technology spillover effects. They provided evidence that the impact of AI on technological innovation varies across sectors, with AI exerting a more significant influence on low-tech sectors in China. This suggests that even countries with limited technological advancement can harness AI to catalyze innovation, particularly in less complex sectors.

### A multidimensional approach

By adopting a multidimensional approach to understand the interaction between scientific research and technological development, various studies have analyzed publication-patent citations [48, 49]. This method involves examining data on citations in local inventors’ patents of scientific publications by researchers in the same region, offering insight into the science-technology relationship. Extensive exploration of this relationship has shown the positive influence of scientific publications on patenting, as evidenced by several studies [31, 50].

However, this approach has limitations, particularly in understanding multidimensionality through paper-patent citation links or volume comparisons. A key challenge is the discrepancy in citation frequency between patents and scientific publications, which hinders the accurate capture of knowledge flow [51]. This indicates that paper-patent citations may not fully capture the comparative advantage in the multidimensional knowledge space among countries. Moreover, a rapid increase in scientific and technological knowledge does not always translate into significant advancements. Recent research suggests stagnation in progress across various fields, with papers and patents becoming less impactful in guiding science and technology in new directions [52]. Thus, comparing the quantity of patents and papers also falls short in attempting to understand spatial spillover.

To address these limitations, Pugliese et al. [15] proposed a novel, multilayered approach to connect scientific, technological, and productive capacity. Expanding on the conditional probability model developed by Hidalgo et al. [32], they devised a tri-layered network to capture interactions among scientific publications, patenting, and industrial production across sectors while incorporating time lags. This method is based on the concept of relatedness, which is the statistically significant co-occurrence of two activities within the same country at a specific time. This framework establishes connections across different activity layers, including scientific fields, technological sectors, and economic production, and offers insight into the capabilities and timelines required to convert technological expertise into economic wealth and scientific innovation.

Catalan et al. [4] furthered this multidimensional approach by introducing the ’cross-proximity’ concept, in which they define scientific and technological cross-density as the average proximity of potential new technologies to a country’s existing scientific and technological portfolio, and examining the influence of endogenous scientific capabilities on technological diversification. Their two-stage method, applied to data from 182 countries over the period of 1988–2014, began with the construction of the ’science and technology cross-space’ network, linking knowledge and technologies based on co-occurrence values. They then assessed the impact of scientific-technological cross-density and technological density on technological diversification at the country level. Their results showed that the proximity of new technology to a country’s scientific portfolio positively affects its probability of adoption. They also discovered that the effect of technological density on diversification surpassed that of scientific and technological density combined.

Moreover, the optimal cognitive proximity theory of the multidimensional space of science and technology suggests that knowledge transfer may be impeded if the cognitive proximity between two entities is either too low or too similar [53]. This is because substantial cognitive distance may impede effective communication, whereas minimal cognitive distance may result in lock-in, preventing learning from external sources.

Despite these advancements, these studies may not have entirely addressed the diverse effects of different knowledge characteristics. They align with traditional economic views that consider technologies to be uniform drivers of economic growth [54, 55]. Conversely, general-purpose technologies such as AI demonstrate high self-productivity, boosting productivity and innovation processes and significantly contributing to economic growth [40, 56–58]. The diffusion potential of AI technologies enables them to permeate a broad and continuously expanding range of applications. Additionally, their complementary nature allows them to augment and enhance products and processes across various industries [58, 59]. This highlights the need to investigate how general-purpose technologies such as AI exhibit complementary effects in a multidimensional space.

## Data and methodology

### Data

#### AI-related patents

The identification of AI technologies is challenging because of the dynamic nature of AI concepts and their broad application across various industries, including autonomous vehicles, drug discovery, and robotics [3, 60]. Consequently, many researchers have proposed diverse methods for extracting information from patents and articles [3, 58, 61–63]. Several strategies for classifying AI technologies have been suggested, such as keyword-based searches of the titles and abstracts of documents such as patents, proceedings, and studies [63, 64]; categorizing AI patents using the Cooperative Patent Classification (CPC) system [62]; and combining key phrases with CPC symbols [61]. These methods can be broadly grouped into key-phrase-based, CPC-based, and hybrid approaches, each with its own advantages and limitations. CPC-based methods are noted for their weak genal-purpose technology (GPT) features such as growth, generality, and complementarity [64], and hybrid approaches exhibit similar characteristics. In contrast, exact keyword matching demonstrates robust growth and generality in technology [64]. In this study, we employed a key-phrase-based method and assessed its robustness by comparing the results with alternative approaches, including the CPC-only method, a combination of keywords and CPC codes, and the use of keywords from titles only. S1 Table in the S1 File provides a detailed overview of these methods.

This study utilizes patents registered with the United States Patent and Trademark Office (USPTO), which we sourced from the European Patent Office’s (EPO) PATSTAT database [65]. The focus on USPTO patents—and the exclusion of patent families from offices such as the Japanese Patent Office (JPO), Korean Intellectual Property Office (KIPO), and China National Intellectual Property Administration (CNIPA)—is due to the unique advantages of the USPTO. First, the USPTO adheres to the Cooperative Patent Classification (CPC) system, a continuously evolving framework that systematically categorizes emerging technologies, including AI, into over 260,000 categories [66]. Second, the lack of a unified classification system for AI-related patent applications across jurisdictions means that each country’s patent management system and standards can lead to data inconsistencies when integrating patents globally [60]. Additionally, patent applicants often file in multiple jurisdictions, potentially leading to duplicate records and overrepresentation of patents from certain countries [60]. Therefore, the USPTO is widely used in research, though it does not encompass innovation activities in all countries [4, 67, 68].

In this study, we collected and refined USPTO patents from the PATSTAT 2022 Spring version, a globally recognized database for bibliographic patent information maintained by the EPO, following specific criteria [65]. AI-related patent applications were initially gathered using AI-relevant keywords from the titles and abstracts of the patent bibliographic information. The approach for selecting AI-related keywords suggested by WIPO [3] was adopted to ensure high relevance to AI (for details on AI-related keywords and CPCs, refer to S1 Table in the S1 File).

When collecting patent applications, we limited the documents to invention patents. Utility models and other non-invention patents were excluded by filtering the PATSTAT ’ipr_type’ field to include only ’PI.’ Applicant and inventor names were sourced from the PATSTAT ’Standardised Name’ field (psn_name), and the country information for applicants and inventors came from the ’person_ctry_code’ field in PATSTAT, with additional processing due to incomplete data coverage. In cases in which a patent inventor was affiliated with institutions in two countries, the patent applications were counted separately for each country without considering the inventor’s proportional contribution. However, no country was assigned when country information was missing. Notably, data for China are underrepresented owing to inconsistencies in applicant and inventor information for Chinese patents in the PATSTAT database, as identified by the UKIPO [60].

After excluding subclasses with no registered patents, 654 subclasses and 407,386 patents were analyzed. To examine temporal patterns, we split the data into nine 5-year snapshots. The data were organized by country (204 countries), time period (eight 5-year intervals), and technology subclass (four-digit CPC codes), resulting in a (204 × 8 × 654) invention patent matrix.

#### AI-related papers

This study analyzed scientific publications from the Web of Science (WoS) Core Collection from 1980 to 2019 to construct a scientific knowledge space. The WoS database includes three primary citation indices: The Science Citation Index Expanded (SCIE), Social Sciences Citation Index (SSCI), and Arts and Humanities Citation Index (AHCI). Our analysis centered on the SCIE sections of the WoS Core Collection, which span five major disciplines: The Arts and Humanities, the Life Sciences and Biomedicine, the Physical Sciences, the Social Sciences, and Technology. In line with the methodology described by Catalan et al. [4], we targeted articles in the SCIE that were directly related to technological inventions in AI. Consequently, this study excluded the SSCI and AHCI sections. Furthermore, we confined our dataset to journal articles and excluded conference proceedings, reviews, and books. Publications lacking an institutional address were also excluded from our analysis.

The WoS bibliographic database enables the classification of papers based on the authors’ institutional affiliations and journal research categories [69]. In this system, publications are associated with countries using the institutional addresses provided by the authors, and each author’s country is considered to contribute equally to the publication. Our study did not distinguish among countries based on the order of authors, the first author, or the degree of co-authorship contribution, though fractional counting and corresponding authorship-based counting are alternative methods. This approach was influenced by WoS’s notably inaccurate coverage of corresponding author information before 2008, which often led to the default of the first author/institution being listed as the corresponding author. Consequently, when an author is affiliated with institutions in multiple countries, journal publications are equally attributed to each of those countries. These methods exhibit high correlations at the macro level [4].

Overall, the WoS is a valuable tool for analyzing the global landscape of AI research. However, potential biases in classifying countries based on author affiliations should be considered. Additionally, it is important to recognize that this method of bibliographic data collection may overestimate research output from Western countries and English-language publications, introducing potential bias in country classification.

Through these meticulous data preprocessing efforts, we constructed our dataset. The AI-related papers we collected covered 230 subcategories out of 252, totaling 468,104 scientific articles published between 1980 and 2019. To examine temporal patterns, we divided the data into eight 5-year intervals. The data were organized by country (170 countries), time period (eight 5-year intervals), and research area (230 disciplines), resulting in a (170 × 8 × 230) matrix of scientific publications.

### Variables

#### Dependent variable

Balassa [20] introduced the RCA to determine a country’s relative advantage for specific products. This methodology can be adapted for specific technologies using the following mathematical representation:

$$RCA_{c,j,t}=\frac{patent_{c,j,t}/\sum_{j}patent_{c,j,t}}{\sum_{c}patent_{c,j,t}/\sum_{c}\sum_{j}patent_{c,j,t}}$$ (1)

Where c, j, and t denote the country, technology, and time, respectively. An RCA value greater than 1 signifies a comparative advantage in relation to technology j for country c at time t. ENTRY refers to the introduction of new technologies or activities into a certain area, such as a city, region, or country. It represents diversification and renewal in technological industries and is used to measure technological changes [28]. In some contexts, ENTRY is used as a binary variable (0 or 1) to indicate the introduction of a new technology with a comparative advantage in a country. The *ENTRY*_*c*,*j*,*t*_ formula represents the probability of a country transitioning to having a comparative advantage in regard to a specific technology (technological specialization). This can be mathematically expressed as follows:

$$ENTRY_{c,j,t}=P(RCA_{c,j,t}>1|RCA_{c,j,t-1}<1)$$ (2)

where *ENTRY*_*c*,*j*,*t*_ calculates the probability that country c will gain a comparative advantage in technology j at time t when it did not have a comparative advantage in regard to the same technology at the previous time point (t − 1). This measure captures the likelihood of a country transitioning from a disadvantaged to advantageous position in a particular technological domain over time. The *EXIT*_*c*,*j*,*t*_ formula denotes the likelihood of a country relinquishing its comparative advantage in a specific technological domain. This is formally defined as follows:

$$EXIT_{c,j,t}=P(RCA_{c,j,t}<1|RCA_{c,j,t-1}>1)$$ (3)

where *EXIT*_*c*,*j*,*t*_ computes the probability that country c loses its comparative advantage in technology j at time t when it possesses a comparative advantage in the same technology during the previous period (t − 1). This metric gauges the propensity of a country to shift from a position of strength to one of weakness in a specific technology field over a given period.

#### Independent variable

*Technology relatedness density*. Technology-relatedness density measures how closely related technologies are clustered around a given technology in a country at a certain time [21]. This can be derived from the technological relatedness of a technology to all other technologies in which the country has a relative technological advantage. This measure is calculated by dividing the sum of the technological relatedness of the technology to all other technologies in a specific country by the sum of the technological relatedness of the technology to all other technologies in a reference country. The calculation process for technology relatedness and technology-relatedness density is as outlined below. First, the technological relatedness (*φ*_*i*,*j*,*t*_) between technologies *i* and *j* is calculated as follows:

$$\varphi_{i,j,t}=P(RCA_{c,j,t}>1|RCA_{c,i,t}>1)$$ (4)

This value denotes the conditional probability that technology *j* exhibits a comparative advantage (RCA > 1) given that technology *i* has an RCA. Second, technology-relatedness density is determined as follows:

$$TECH\_DEN_{c,j,t}=\frac{\sum_{j\in c,j\neq i}\varphi_{i,j,t}}{\sum_{i\neq j}\varphi_{i,j,t}}\times 100$$ (5)

This metric measures the average degree of technological relatedness between technology *i* and all other technologies *j* in country *c* at time *t*, excluding *i*. The value of relatedness density ranges from 0% to 100%. A value of 0% indicates that there is no technology related to technology i in the country in question, whereas a value of 100% indicates that all technologies related to technology i belong to the country’s technological portfolio.

*Science-technology cross-proximity density*. The cross-proximity density between science and technology, also known as ‘sci-tech cross-density,’ is a measure of how the scientific development in a country is connected to the technology sectors in which it aims to grow [15]. This measure indicates the average proximity of a technology class to a country’s current scientific structure during a specific period. A high sci-tech cross-density value implies that a country has a high degree of scientific development in areas closely related to the technology field that it aims to develop. The importance of this measure lies in its ability to reveal a country’s potential to develop new technologies based on its existing scientific capabilities [4].

For the cross-density analysis, we first calculated the proximity between scientific fields and technological classes. This calculation reflects the extent of the overlap or connection between scientific fields and technological classes within a specific context, such as a country. This is instrumental in revealing how a country’s scientific capabilities align with those of its technological class. A high proximity value indicates a close relationship between a country’s scientific research and the technological areas on which it is focusing, potentially leading to more efficient technological development and innovation. This proximity was measured using the minimum conditional probability, a well-established method in the literature [4, 15, 32]. This approach selects pairs of scientific fields and technological domains that both exhibit an RCA greater than one. For these pairs, the minimum conditional probability is determined using the lower of the two calculated conditional probabilities as a cross-proximity measure. This conservative approach offers a more accurate estimate of the relatedness between the two entities. The formula used for this measurement is as follows:

$$\varphi_{sci,tech,t}^{X}=\min\left\{\begin{array}{l} P(RCA_{sci,t}>1|RCA_{tech,t}>1)\\ P(RCA_{tech,t}>1|RCA_{sci,t}>1) \end{array}\right\}$$ (6)

where sci denotes the scientific category, tech is the technological field, and t is the period. Second, to measure cross-relatedness density, we computed the average sci-tech cross-proximity for a technology class relative to the country’s existing scientific structure within a specified time period. This was achieved using the following equation:

$$CROSS\_DENSITY=\omega_{c,tech,t}^{X}=\frac{\sum_{tech\in c,tech\neq sci}\varphi_{sci,tech,t}^{X}}{\sum_{sci\neq tech}\varphi_{sci,tech,t}^{X}}\times 100$$ (7)

In this equation, cross-density quantifies the average degree of cross-proximity between a specific technological class (e.g., as defined by a four-digit CPC code) and all other scientific fields in Country c at time t. This metric is essential, as it reveals the interrelatedness and co-occurrence patterns between scientific and technological fields, providing insight into the dynamics in a country’s innovation ecosystem.

*Technology complexity*. The technology complexity index (TCI) quantifies the complexity of technologies by analyzing the structure of the bipartite network that links countries to the technologies they develop. We employ the ’Method of Reflections’ technique, as proposed by Hidalgo and Hausmann [33], to calculate technology complexity. This approach accounts for a country’s diversity (the range of technological fields it produces) and the ubiquity of the technologies in question (how commonly they are found in different countries). Initially, a country’s diversity is determined by the number of technologies it produces, and the ubiquity of a technology is indicated by the number of countries producing it. Diversity measures the degree of centrality of country *c* in a bipartite network linking countries to technologies:

$$DIVERSITY=\sum_{j}M_{c,j}$$ (8)

The ubiquity represents the degree centrality of technological class *j*:

$$UBIQUITY=\sum_{c}M_{c,j}$$ (9)

Here, *M*_*c*,*j*_ refers to a two-mode matrix involving country c and technology j. It is defined as follows:

$$M_{c,j}\;(j=1\;if\;RCA\geq 1,\;otherwise\;0)$$ (10)

This matrix indicates whether country *c* has a comparative advantage (RCA) in the production of technology *j*. In this approach, the use of ‘*M*_*c*,*j*_’ effectively minimizes undue variation by focusing solely on a marked presence (*M*_*c*,*j*_ = 1) or absence (*M*_*c*,*j*_ = 0). In the second step, technology complexity is calculated by sequentially combining diversity and ubiquity using two equations over a series of *n* iterations:

$$ECI=\frac{1}{UBIQUITY}\sum M_{c,j}TCI$$ (11)

$$TCI=\frac{1}{DIVERSITY}\sum M_{c,j}ECI$$ (12)

where ECI represents the economic complexity index, TCI signifies the technology complexity index, c denotes a country, and j denotes a technology. With each subsequent iteration, this method yields increasingly precise estimates of complexity by integrating feedback effects and considering the complexity of the technologies produced by a country or that of countries producing a particular technology. The iterations cease when the ranking of countries and technologies stabilizes from one step to the next.

### Regression model

To examine the probability of binary dependent variables, namely the entry and exit of technology, this study employs a panel logit model incorporating three-way fixed effects (FEs) for country, technology, and time to account for unobserved, time-invariant heterogeneity across cross-sectional units in countries (Country FEs), technologies (CPC FEs), and periods (Period FEs).

The specified model is as follows:

$$\begin{array}{l} ENTRY_{c,j,t}=\beta_{1}\times TECH\_DENSITY_{c,j,t-1}+\beta_{2}\times TECH\_COMPLEXITY_{j,t-1}\\ +\beta_{3}\times CROSS\_DENSITY_{c,j,t-3}+\beta_{4}\times TECH\_DENSITY_{c,j,t-1}^{sq}\\ +\beta_{5}\times TECH\_COMPLEXITY_{j,t-1}^{sq}+\beta_{6}\times CROSS\_DENSITY_{c,j,t-3}^{sq}\\ +\beta_{7-10}\times CONTROL_{c,j,t-1}+\alpha_{c}+\gamma_{j}+\delta_{t}+\varepsilon_{c,j,t} \end{array}$$ (13)

where *c*, *j*, and *t* represent the country, technology, and time period, respectively. The dependent variables include ENTRY and EXIT, which represent the introduction and termination of the technology, respectively. In the ENTRY model, the dependent variable is assigned a value of 1 if country *c* adopts a new RCA technology at time *t*; otherwise, the value is 0. In the EXIT model, the dependent variable takes a value of 1 if country c discontinues a preexisting RCA technology at time *t* and 0 otherwise. The independent variables of technology relatedness density (TECH_DENSITY) and technology complexity (TECH_COMPLEXITY) are lagged by 1 year, whereas sci-tech cross-density (CROSS_DENSITY) is lagged by 3 years, given that scientific knowledge adoption may take 3 years. Although previous studies [4] considered 4-year lag periods, the adoption process in AI fields, particularly computer science, may be faster [3]. We also tested the robustness by changing the time lag from 0 to 4. Because complexity and density may have an inverted U-shaped relationship with the dependent variable, this study included squared terms for the independent variables. The control variables (POP, GDP_CAPITA, TECH_STOCK, and TECH_SIZE) were logarithmically transformed to mitigate size effects and normalize their distribution. As outlined in S2 Table of S1 File, all of the control variables are lagged by 1 year. To account for FEs, this study included *α*_*c*_, *γ*_*j*_, and *δ*_*t*_, which represent the country, technology, and time, respectively. In the preceding model, *ε*_*c*,*j*,*t*_ represents the error term that captures the unexplained variation in the dependent variable that is not accounted for by the included independent variables and FEs.

## Results

### Descriptive statistics

Table 1 presents the variables’ descriptive statistics and correlation coefficients. The key independent variables (TECH_DENSITY, TECH_COMPLEXITY, and CROSS_DENSITY) have values ranging from 0 to 100. Because of the requirement for simultaneous observations in the science–technology space, TECH_DENSITY and TECH_COMPLEXITY had 26,300 observations each, whereas CROSS_DENSITY had 19,035 observations. Values observed only in the technology or science space were removed. The number of observations for the control variables varies due to uneven data collection across countries for TECH_SIZE, which also has 26,300 observations representing patents per CPC. The correlation coefficients between the variables were generally low.

**Table 1:** Descriptive statistics and correlations.

Variables		1	2	3	4	5	6	7	Obs.	Min	Max	Mean	SD
1	TECH_DENSITY	1.0							26,300	-6.08	93.91	-1.51e-07	11.29
2	TECH_COMPLEXITY	–0.243	1.0						26,300	-60.63	39.36	1.45e-06	30.89
3	CROSS_DENSITY	0.531	–0.044	1.0					19,035	-22.46	77.53	3.22e-07	18.16
4	POP	0.278	0.002	0.306	1.0				24,719	9.62	21.05	15.92	2.28
5	GDP_CAPITA	0.327	–0.025	0.501	–0.413	1.0			23,583	5.63	12.08	9.09	1.36
6	TECH_STOCK	0.582	0.007	0.609	0.322	0.518	1.0		25,754	0.69	14.77	7.47	2.77
7	TECH_SIZE	0.082	–0.334	0.116	–0.044	0.063	–0.067	1.0	26,300	2.39	12.82	9.61	1.35

Note: TECH_DENSITY, TECH_COMPLEXITY, and CROSS_DENSITY are mean-centered. SD refers standard deviation.

**Fig 1:**
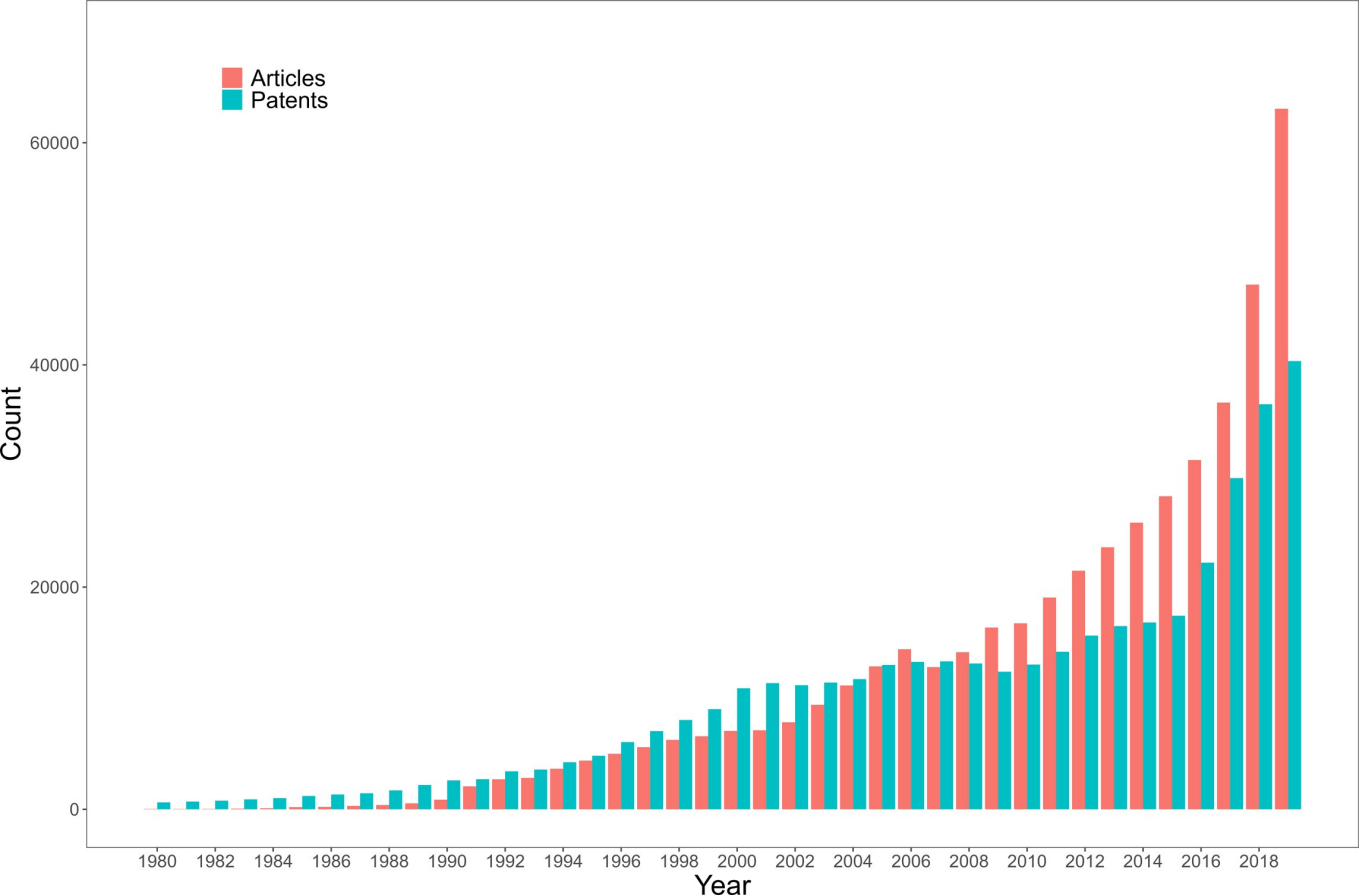
Number of AI articles and patents by year.

Fig 1 presents the trends in AI research and patenting activities represented by the annual number of AI-related patents and articles from 1980 to 2019 in global countries. This overview delineates the expansion and progression of AI-related research and innovation. The uninterrupted augmentation of AI-centered innovation activities in both scientific and technological sectors has been discernible since the 1980s. Notably, during the 2010s, the proliferation of research publications outpaced that of patents, emphasizing the accelerating interest in AI research and development across diverse disciplines.

Table 2 presents the top countries in AI knowledge production, which encompasses both technological and scientific knowledge, and the corresponding fields from 1980 to 2019. In addition, the top 10 countries, CPC symbols, and disciplines were identified based on the highest number of patents and articles.

**Table 2:** Top AI knowledge production countries and fields, 1980–2019.

Technological Knowledge				Scientific Knowledge			
Country	Patents per country	CPCs	Patents per CPC	Country	Articles per country	Disciplines	Articles per discipline
US	202,867	G06F	78,504	US	115,120	Computer Science (AI)	84,501
KR	48,915	G06N	71,052	CN	114,944	Engineering (Electrical & Electronics)	75,808
JP	36,548	H04L	38,946	UK	39,246	Computer Science (Interdisciplinary)	34,659
DE	35,995	G06Q	38,050	GE	26,235	Computer Science (Information Systems)	34,287
CN	32,102	G06V	31,098	CA	23,667	Neuroscience	33,175
FR	14,807	G06T	30,236	IN	21,776	Computer Science (Theory & Method)	28,257
CA	13,917	G06K	28,880	SE	19,836	Automation and Control Systems	20,353
UK	13,197	H04W	22,693	IT	19,817	Operations Research & Management Science	18,525
NL	9,542	H04N	20,235	IR	19,590	Environmental Sciences	17,066
IN	8,374	A61B	18,880	FR	19,035	Telecommunications	16,534

In terms of technological knowledge, the United States had 202,867 patents, followed by South Korea, Japan, Germany, and China. The most prevalent CPCs were found to be G06F, G06N, H04L, G06Q, and G06V. The United States ranked first in scientific knowledge with 115,120 articles, followed by China, the United Kingdom, Germany, and Canada. The top disciplines in scientific knowledge production were computer science (AI), engineering (electrical and electronic), computer science (interdisciplinary), computer science (information systems), and neuroscience.

### Science–technology space

In this study, we employ co-occurrence-based knowledge networks to probe the interaction between scientific and technological knowledge generation activities, emphasizing the period following the rapid AI advancements of the 2010s. The significant strides made in AI since 2012, underscored by the success of AlexNet [44], provide a context for exploring the science–technology nexus.

**Fig 2:**
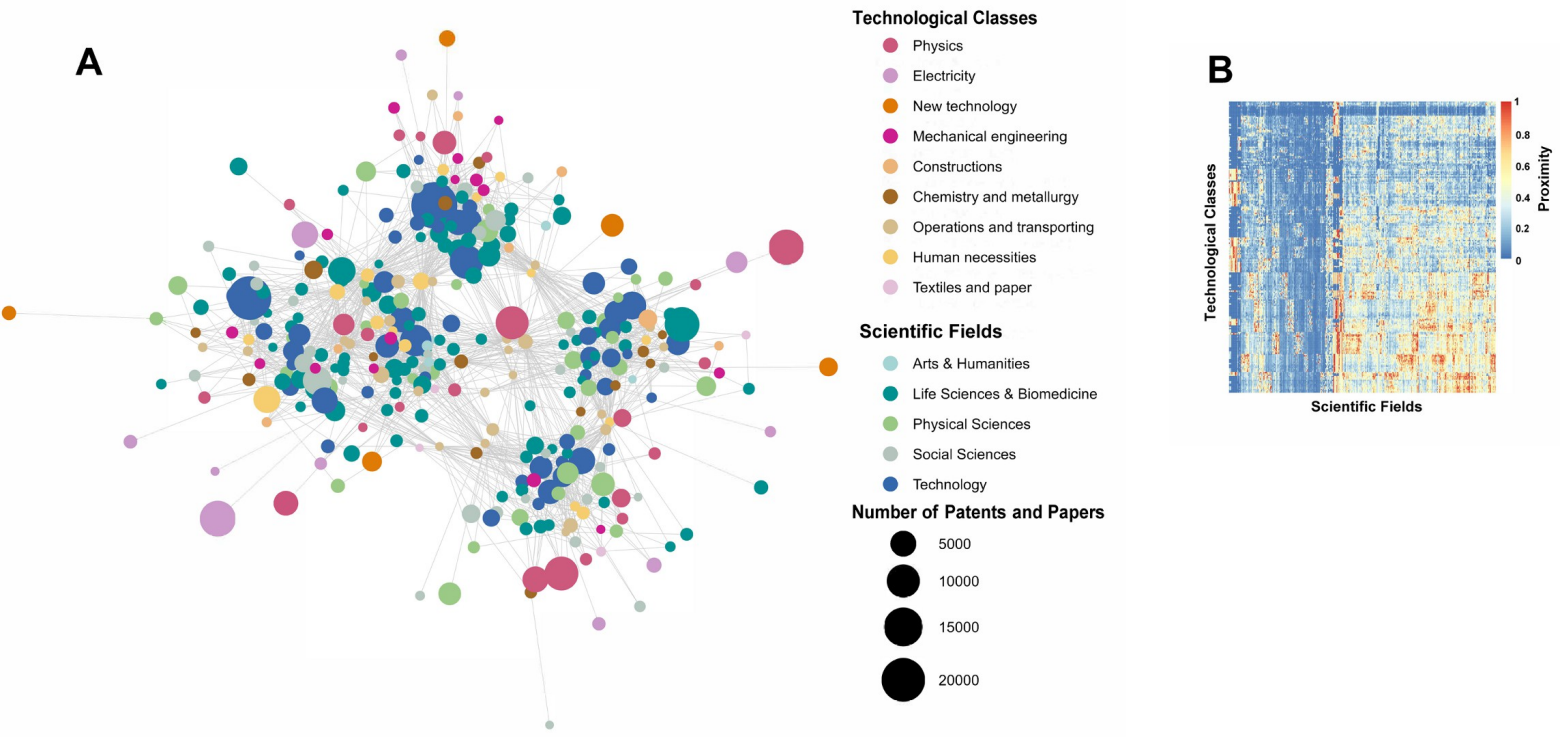
Science–technology knowledge space. (A) Visualization of the science–technology intersection space. (B) Representation of the hierarchically clustered proximity matrix.

**Fig 3:**
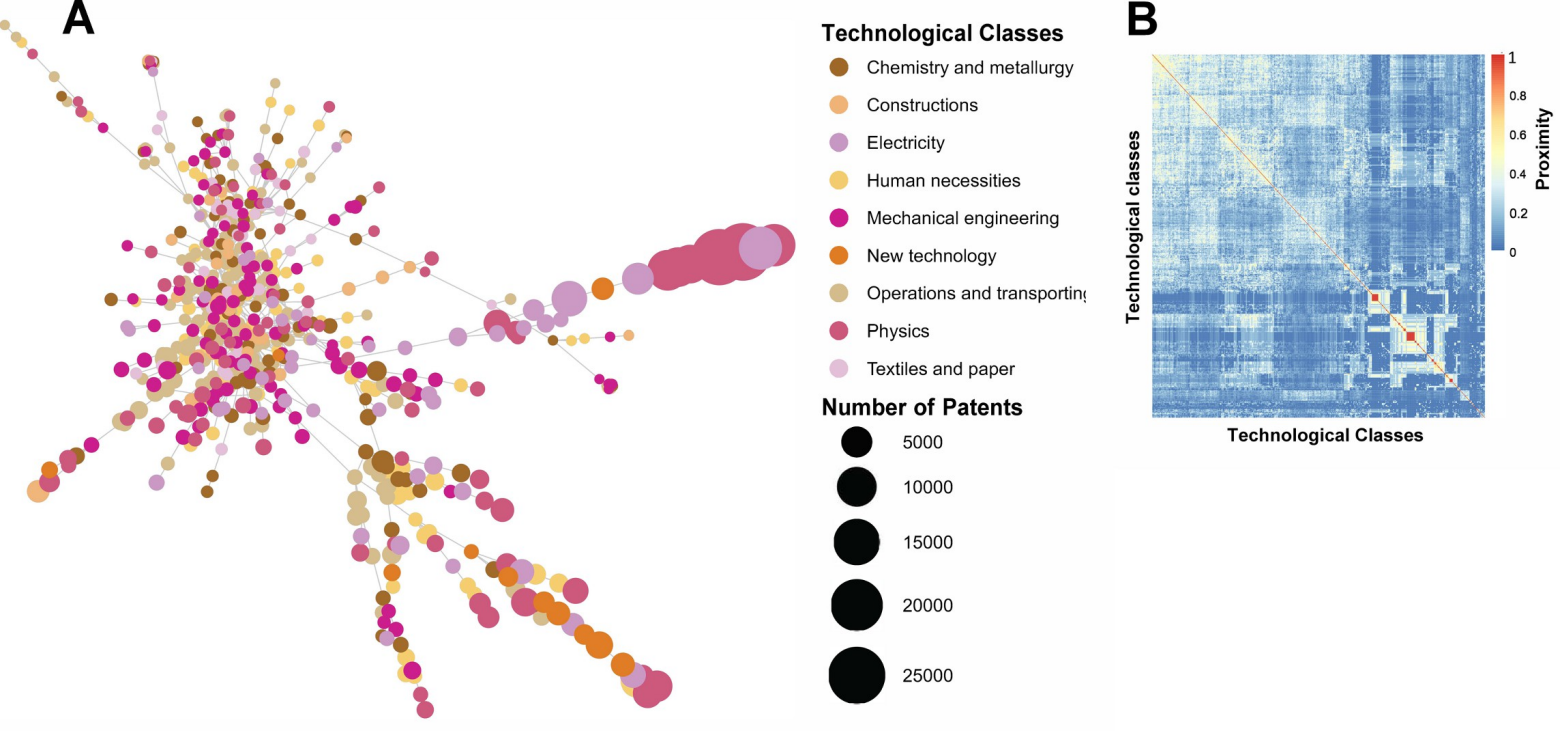
Scientific knowledge space. (A) Graphic representation of the science space. (B) Hierarchically clustered proximity matrix depiction.

**Fig 4:**
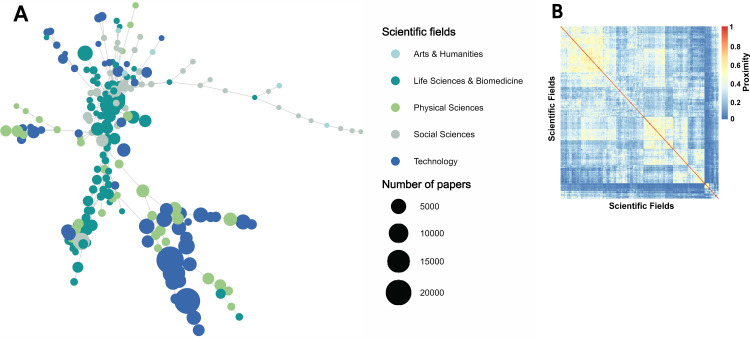
Technological knowledge space. (A) Graphical illustration of the technology space. (B) Hierarchically clustered proximity matrix representation.

Fig 2 depicts a knowledge space with 161 scientific areas and 145 CPCs across 112 countries from 2013 to 2017. This space, the product of the matrix in Fig 3B and its transposed version in Fig 4B, illustrates the degree of interaction between science and technology. As shown in Fig 2A, a network layout algorithm [70] situates undirected graph nodes based on their pairwise distances. The size of each node signifies the patent and article counts per CPC and discipline, with the labels representing the initial three digits of each discipline and CPC category. These were subdivided into five scientific and eight technological fields. Fig 2B presents the cross-proximity matrix clustering of the scientific areas and technological fields with high co-occurrence probabilities. A limited number of these fields and areas, particularly G06, H04, and A61 (as indicated in Table 2), are significantly involved in the production of AI knowledge, including prolific disciplines such as computer science.

Fig 3 illustrates the science space, encompassing 225 scientific areas in 160 countries from 2013 to 2017. The WoS Core Collection [69], a widely used database for scholarly research, was used to categorize these scientific areas. This figure depicts the distribution of the scientific areas and highlights the most active and visible research areas. Fig 4 shows the technology space, which includes 630 four-digit CPCs from 144 countries during the same period. The CPC scheme is divided into nine sections (A–H and Y) and further subdivided into classes, subclasses, groups, and subgroups. This figure offers a comprehensive view of technological fields, highlighting the most productive research and development (R&D) areas. Collectively, these figures contribute to a broad understanding of the science–technology space, making it easier to identify potential opportunities for academia–industry collaboration and future research.

### Panel logit regression results

Table 3 presents the panel logit regression results for ENTRY, examining three primary independent variables (TECH_COMPLEXITY, TECH_DENSITY, and CROSS_DENSITY) as well as control variables. In the panel logit model, the coefficients represent the estimated changes in the log odds of the outcome for a one-unit increase in the predictor variable. Therefore, these coefficients do not denote the direct probabilities. Instead, they are associated with odds, defined as the ratio of an event’s probability (in this context, ENTRY) to the non-occurrence of the same event. Odds ratios are obtained by exponentiating logistic regression coefficients. For example, the coefficient of TECH_DENSITY in Table 3 is 0.3206. The corresponding odds ratio, calculated as e^0.3206^, equates to 1.378, suggesting a 37.8% increase in the odds of ENTRY for each unit increment in TECH_DENSITY. The findings reveal a statistically significant relationship between entry and both technology complexity and relatedness density at the 1% level. Specifically, increases in these variables are associated with a greater likelihood of entry into a new technology when the other variables are held constant.

**Table 3:** Panel logit regression results.

	Dependent variable: ENTRY				Dependent variable: EXIT			
	M1	M2	M3	M4	M1	M2	M3	M4
TECH_DENSITY	0.2137*** (0.0031)	0.3225*** (0.0047)	0.2137*** (0.0031)	0.3206*** (0.0047)	–0.1019*** (0.0031)	–0.1800*** (0.0067)	–0.1019*** (0.0031)	–0.1802*** (0.0068)
TECH_COMPLEXITY	0.0073*** (0.0015)	0.0135*** (0.0015)	0.0066*** (0.0015)	0.0142*** (0.0016)	–0.0096*** (0.0031)	–0.0131*** (0.0031)	–0.0096*** (0.0031)	–0.0130*** (0.0032)
CROSS_DENSITY	0.0320*** (0.0016)	0.0286*** (0.0015)	0.0319*** (0.0016)	0.0431*** (0.0022)	0.0049** (0.0022)	0.0048** (0.0021)	0.0049** (0.0022)	0.0057 (0.0045)
DENSITY_sq		–0.0024*** (0.0001)		–0.0023*** (0.0001)		0.0011*** (0.0001)		0.0011*** (0.0001)
TECH_COMPLEXITY_sq			–0.0001** (0.0001)	–0.0001** (0.0001)			0.0000 (0.0001)	0.0000 (0.0001)
CROSS_DENSITY_sq				–0.0004*** (0.0001)				–0.0000 (0.0001)
POP	1.6318*** (0.1948)	1.5062*** (0.1980)	1.6182*** (0.1949)	1.3460*** (0.2012)	0.4190 (0.6125)	0.5112 (0.6204)	0.4204 (0.6129)	0.5152 (0.6208)
GDP_CAPITA	0.5374*** (0.0700)	0.5041*** (0.0713)	0.5289*** (0.0701)	0.4381*** (0.0719)	0.3316* (0.1742)	0.3216* (0.1825)	0.3318* (0.1743)	0.3240* (0.1826)
TECH_STOCK	–0.1529*** (0.0391)	–0.3956*** (0.0406)	–0.1492*** (0.0391)	–0.4172*** (0.0407)	0.4199*** (0.0985)	0.5690*** (0.1033)	0.4198*** (0.0985)	0.5682*** (0.1033)
TECH_SIZE	0.4954*** (0.0413)	0.4721*** (0.0417)	0.4951*** (0.0412)	0.4585*** (0.0418)	–0.3353*** (0.0858)	–0.3212*** (0.0867)	–0.3354*** (0.0858)	–0.3216*** (0.0868)
Country FEs	YES	YES	YES	YES	YES	YES	YES	YES
CPC FEs	YES	YES	YES	YES	YES	YES	YES	YES
Period FEs	YES	YES	YES	YES	YES	YES	YES	YES
N (group)	55,154 (11,403)	55,154 (11,403)	55,154 (11,403)	55,154 (11,403)	9,691 (3,059)	9,691 (3,059)	9,691 (3,059)	9,691 (3,059)
LR (χ^2)	17615***	17778***	17620***	18703***	2096***	2273***	2096***	2274***

Notes: Standard errors in parentheses

* p < 0.1,

** p < 0.05,

*** p < 0.01

The dependent variables (ENTRY or EXIT) are binary (0 or 1). The independent and control variables are lagged by 1 year except for CROSS_DENSITY, which is lagged by 3 years. The term ’sq’ indicates a squared term.

Moreover, science–technology cross-density demonstrates a positive and statistically significant association with ENTRY at the 1% level, indicating that increased proximity to a country’s scientific and technological portfolio increases the probability of technology entry when controlling for other factors. The effects on the likelihood of ENTRY becoming 1 were most significant in the order of TECH_DENSITY, CROSS_DENSITY, and TECH_COMPLEXITY. The squared terms of the primary independent variables display negative and statistically significant relationships with ENTRY, signifying possible diminishing returns or complex dynamics. Furthermore, as shown in S3 Fig of S1 File, the combined effect of technological complexity and scientific–technological relatedness density leads to an ENTRY probability of 1. Thus, we can infer that the optimal combination of knowledge complexity and scientific–technological collaboration maximizes the effect of technological diversification in a given country.

In models in which EXIT is the dependent variable, the coefficients represent the impact of the independent variables on the logarithmic odds of a technology’s exit from a country. The analysis revealed that increases in technology complexity and technology-relatedness density are linked to a decreased likelihood of technology exit at the 1% significance level. Science-technology cross-density exhibits a positive association with EXIT at the 5% significance level in specific models; however, this relationship becomes statistically insignificant in subsequent models. The squared terms suggest a nonlinear relationship between technology-relatedness density and EXIT, whereas the relationships involving technological complexity and cross-density remain ambiguous. In conclusion, the results indicate distinct associations between the main independent variables and technology exit compared to technology entry. These findings have considerable implications for understanding the factors that influence entry and exit, ultimately informing policymaking related to innovation.

### Robustness checks

Table 4 presents the robustness assessments of the main findings of Model 4 for ENTRY and EXIT, focusing on the independent variables segmented by income level and the top 10 AI knowledge producers. Key insights include the top 10 countries leveraging more complex knowledge for entry, as indicated by the greater effects of technology complexity compared to other countries, but showing lower technology-relatedness density effects. This suggests a reliance on advanced knowledge over path-dependent preexisting knowledge. Similar patterns emerge across income groups, with high-income countries displaying greater technology complexity effects on ENTRY, underscoring the varying effects of technology complexity and relatedness density in diverse contexts.

**Table 4:** Panel logit regression results (outcome level).

	Dependent variable: ENTRY				Dependent variable: EXIT			
	High	Mid and Low	TOP10	Others	High	Mid and Low	TOP10	Others
TECH_DENSITY	0.3012*** (0.0048)	0.3985*** (0.0130)	0.1771*** (0.0039)	0.4135*** (0.0073)	–0.1715*** (0.0066)	–0.2049*** (0.0458)	–0.1236*** (0.0052)	–0.2745*** (0.0166)
TECH_COMPLEXITY	0.0196*** (0.0020)	0.0072** (0.0031)	0.0314*** (0.0030)	0.0106*** (0.0021)	–0.0148*** (0.0039)	–0.0074 (0.0105)	–0.0227*** (0.0049)	–0.0046 (0.0064)
CROSS_DENSITY	0.0308*** (0.0022)	0.0771*** (0.0060)	0.0155*** (0.0023)	0.0653*** (0.0033)	0.0053 (0.0040)	–0.0032 (0.0308)	0.0039 (0.0037)	0.0078 (0.0089)
DENSITY_sq	–0.0023*** (0.0001)	–0.0032*** (0.0002)	–0.0015*** (0.0001)	–0.0033*** (0.0001)	0.0011*** (0.0001)	0.0004 (0.0009)	0.0010*** (0.0001)	0.0026*** (0.0003)
TECH_COMPLEXITY_sq	–0.0001*** (0.0001)	–0.0001 (0.0001)	–0.0001 (0.0001)	–0.0001** (0.0001)	0.0001 (0.0001)	–0.0005 (0.0002)	0.0001 (0.0001)	0.0002* (0.0001)
CROSS_DENSITY_sq	-0.0002*** (0.0001)	-0.0010*** (0.0002)	-0.0001 (0.0001)	-0.0006*** (0.0001)	-0.0001 (0.0001)	0.0001 (0.0008)	0.0001 (0.0001)	-0.0002 (0.0001)
POP	1.0770*** (0.2502)	0.5745 (0.4220)	1.6070** (0.7042)	1.6911*** (0.2331)	0.18055 (0.7088)	–2.9284 (1.9322)	0.4967 (1.1141)	–0.6695 (0.8257)
GDP_CAPITA	0.7627*** (0.1108)	0.2326* (0.1333)	–0.1816 (0.1470)	0.9308*** (0.0932)	0.4229 (0.2717)	–0.1204 (0.4091)	–0.0753 (0.2843)	0.2992 (0.2766)
TECH_STOCK	–0.3366*** (0.0575)	–0.5412*** (0.0802)	–0.3277*** (0.0853)	–0.4470*** (0.0523)	0.6956*** (0.1294)	0.2322 (0.2448)	0.8476*** (0.1676)	0.4627*** (0.1471)
TECH_SIZE	0.4049*** (0.0489)	0.5592*** (0.0861)	0.2531*** (0.0726)	0.5349*** (0.0529)	–0.3441*** (0.0914)	–0.2729 (0.3124)	–0.3343*** (0.1200)	–0.3602*** (0.1354)
Country FEs	YES	YES	YES	YES	YES	YES	YES	YES
CPC FEs	YES	YES	YES	YES	YES	YES	YES	YES
Period FEs	YES	YES	YES	YES	YES	YES	YES	YES
N (group)	41,153 (8,284)	13,496 (3,027)	16,730 (3,530)	37,726 (7,794)	8,673 (2,639)	1,004 (414)	5,489 (1,587)	4,191 (1,471)
LR (χ^2)	14482***	4388***	6331***	12903***	2107***	217***	1542***	849***

Notes: Standard errors in parentheses

* p < 0.1,

** p < 0.05,

*** p < 0.01

The dependent variables (ENTRY and EXIT) were employed from Model 4 in the main results (Table 3). High denotes a high-income country. Middle- and low-income countries encompass upper-middle-, lower-middle-, and low-income countries. TOP10 denotes the top 10 countries that are most productive in terms of patents, whereas “Others” denotes the remaining countries.

The EXIT models demonstrate a negative correlation between technology complexity and EXIT across all groups, with negative effects being more pronounced in technologically advanced countries. This finding indicates that increased technological complexity reduces the probability of industry exit in these countries. Similarly, technological relatedness density negatively influences EXIT across all groups, suggesting that increased relatedness density decreases the odds of industry exit. Nonetheless, the effects of scientific and technological relatedness density on EXIT are not statistically significant, indicating that the interaction between science and technology does not contribute significantly to a nation’s ability to maintain technological competitiveness. Despite the differences in income levels and top AI knowledge producers compared to the average, the relationships were statistically significant and consistent with expectations: technologically advanced countries tend to capitalize on complex knowledge, lowering the likelihood of exit from a technologically complex industry.

Conversely, less advanced countries appear to rely more on preexisting knowledge bases, exhibiting distinct technology entry and exit dynamics compared to the global norm. These findings add to our understanding of the determinants of technology entry and exit and may have implications for future innovation policy formulation while preserving the robustness of the findings. The notion that income-specific factors shape these relationships suggests that policymakers should further investigate these relationships.

To improve the reliability and consistency of our findings, we conducted robustness checks on the panel logit models using various specifications, accounting for potential biases and confounding influences. This involved stratifying the specifications by grouping and applying three-way FEs for countries, technologies, and periods. Initially, the countries were categorized by income level, which facilitated the validation of our results at different stages of development and the exploration of heterogeneity between income groups. Subsequently, the countries were divided into two groups: the top 10 countries that produced the most knowledge in terms of technological advancement and the remaining countries. This allowed us to examine whether the level of technological development in a country influences the regression model coefficients. Technologies were classified according to specific schemes to address the potential effects of technology-specific characteristics on the relationships between the dependent and independent variables. This step helped ensure the robustness of our findings across various technologies. Finally, time lags were incorporated into the analysis to control for potential period-specific effects, recognizing that relationships between variables might evolve or display time-lagged effects and strengthening the robustness and consistency of our results across time frames.

In conclusion, when countries were divided into groups based on their economic and technological maturity, deviations from the global average were observed. Nonetheless, the primary model’s main findings retained statistical consistency and significance, as shown in Table 4.

For additional validation, S4 Table in S1 File provides a robustness check of Model 4 from Table 3 using various AI classification schemes: 1) key phrases from patent document abstracts and titles, 2) key phrases from patent document titles, and 3) CPC symbols as suggested by the World Intellectual Property Organization. Despite some differences among these schemes and the limitations of the CPC-only approach, the robustness of the relationships between the independent and control variables as well as ENTRY and EXIT was confirmed across the schemes, underscoring the validity and generalizability of the main results.

S5 Table in S1 File evaluates the robustness of the main findings by varying the number of lagged years for ENTRY in Model 4 from Table 3. The analysis covering lags 0 to 4 reveals a consistently positive and statistically significant relationship with ENTRY across all models, thereby verifying the robustness of the main findings over different time lags. S6 Table in S1 File presents a similar robustness test for EXIT. Again, the results reveal consistent relationships among technological complexity, density, and EXIT. However, the relationship between scientific proximity and EXIT is less consistent, indicating potential time-lagged effects or nuances that warrant further investigation.

### Marginal and net effect analysis

**Fig 5:**
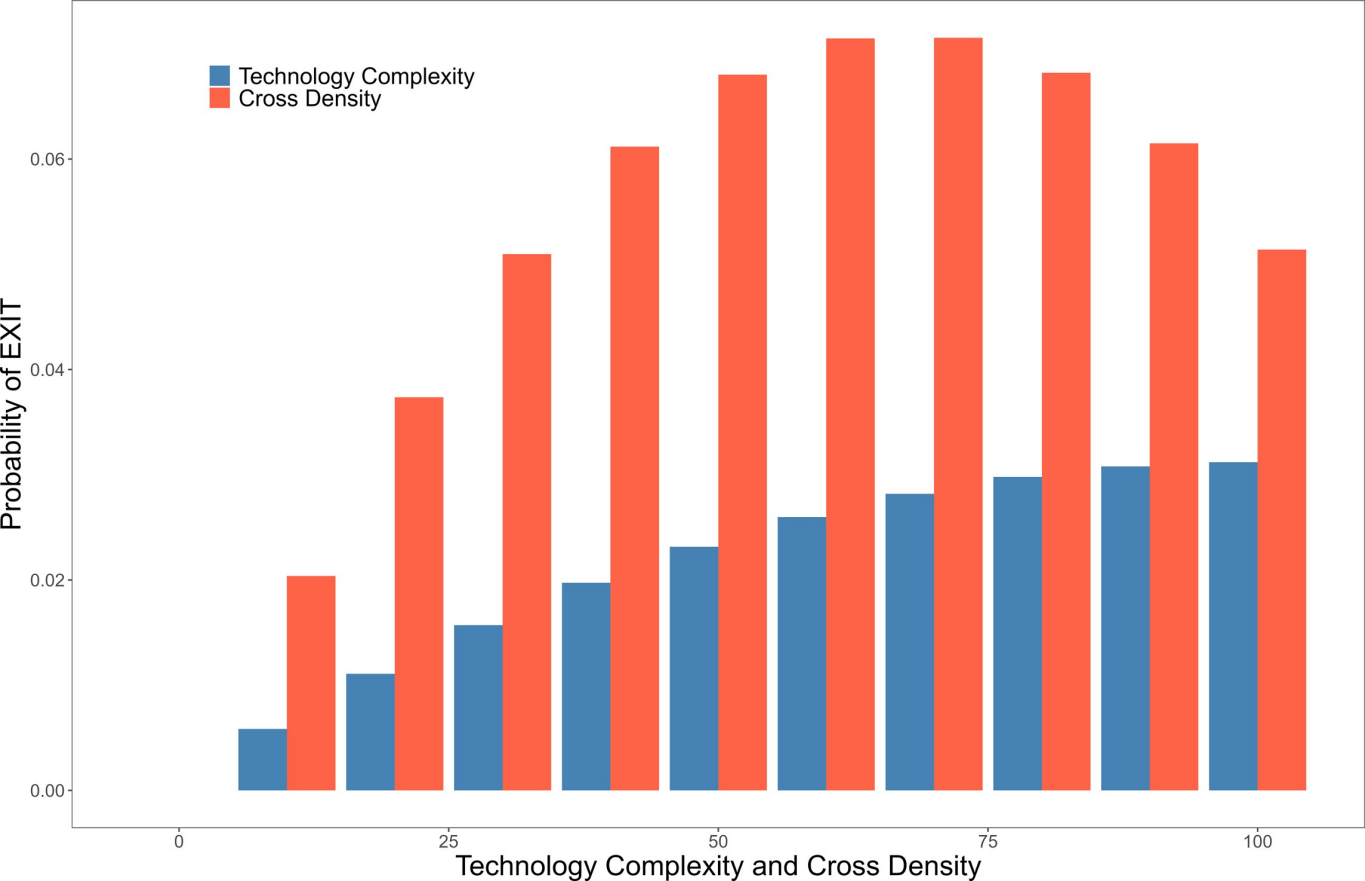
Marginal effects of technology complexity and cross-density on the probability of ENTRY.

**Fig 6:**
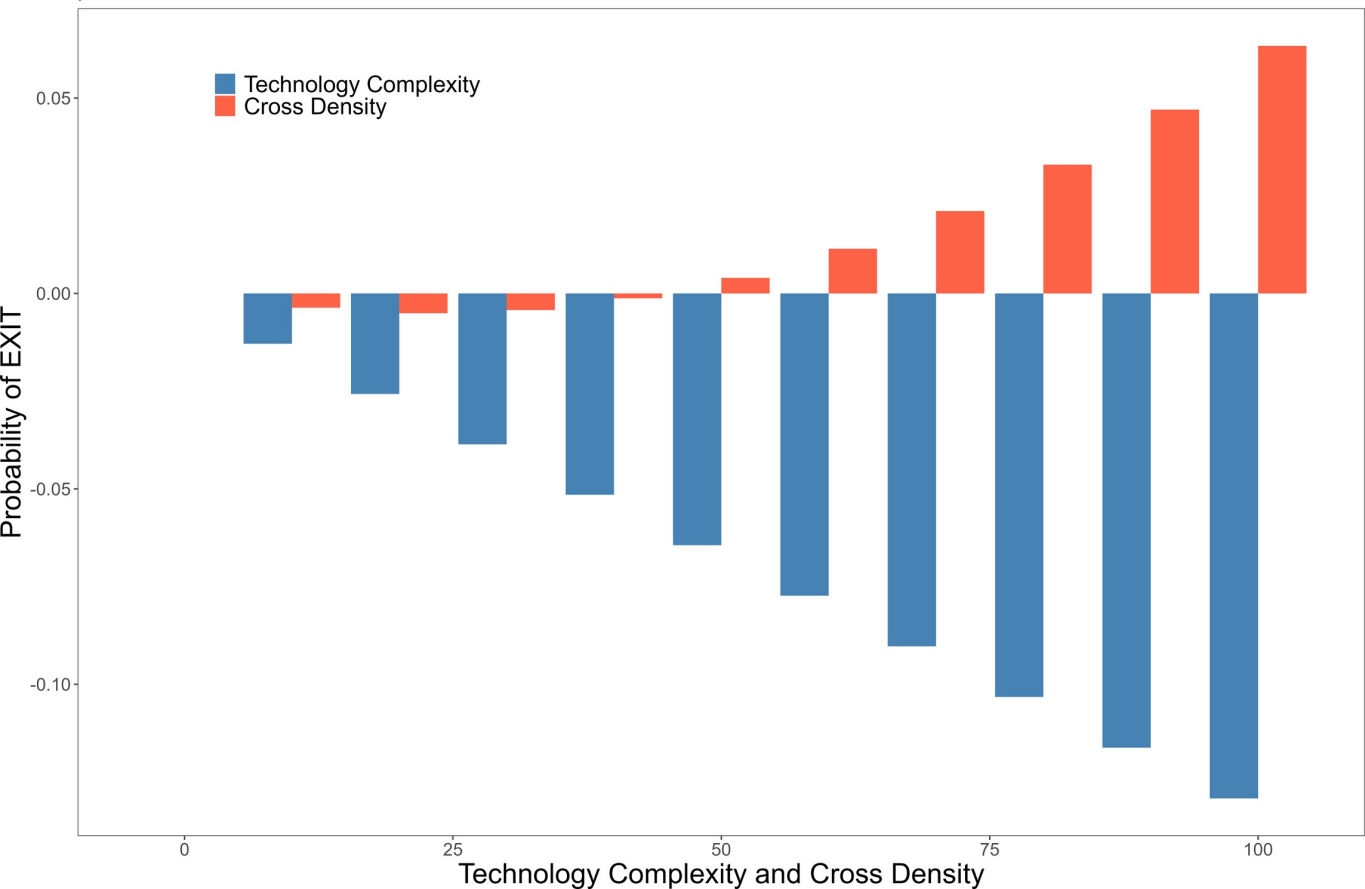
Marginal effects of technology complexity and cross-density on the probability of EXIT.

To elucidate the relationship between AI technology dynamics and influential factors, we present the estimated average marginal effects of these factors on the probability of AI technology entry and exit (S7 Table in S1 File). This study estimated the average partial effects for binary regression models with FEs and identified significant coefficient values at the 95% level or higher. As shown in Fig 5, the average marginal effect of cross-density on ENTRY exceeds that of technological complexity. These factors are positively correlated with a country’s AI technology specialization. Fig 6 shows the average marginal effects of technology complexity and science–technology cross-relatedness density on the probability of AI technology exit. Both of these factors attenuate the potential of AI technology to exit a country up to a certain threshold, beyond which they have divergent effects. The probability of AI technology exit diminishes with the escalation of technology complexity. However, cross-density that surpasses a certain threshold fails to safeguard against the exit of AI technologies.

Furthermore, the net effects were calculated for quadratic terms that had statistically significant marginal effects at the 95% level or higher. For example, in S7 Table’s Model 2 of S1 File, the net influence of technological relatedness on ENTRY is 0.01045, derived from 2 × [–0.00007 × –0.000000151] + [0.0104588], with an average value of -0.000000151, a marginal impact of –0.00007, an unconditional effect of 0.0104588, and a factor of 2 from the quadratic derivation. The directionality and statistical significance of these coefficients are corroborated in the framework of the linear probability model, albeit with varying degrees of influence.

Hence, the results indicate that although the synergistic effects of scientific knowledge and technology contribute considerably to creating new knowledge for competitive advantage, complex technologies exert a weaker influence on new knowledge creation but sustain a potent relationship with retaining competitiveness in extant technologies.

## Discussion and conclusions

This study examined the determinants of AI technology specialization, a key element of national competitiveness. We evaluated technological specialization at the country level using an RCA-based method to quantify the relative advantages of technologies across multiple countries. In particular, our study introduced the concept of cross-proximity density to explore how scientific research underpins technological progress in AI. Employing a three-way fixed-effects panel logit model, we analyzed data from 170 countries over the period of 1980 to 2019.

Building on this framework, we discovered that AI specialization is path dependent. This finding implies that a country’s transition to new AI technologies is heavily influenced by its existing technological capabilities. Countries tend to specialize in AI technologies related to their pre-existing technological portfolios, supporting the principle of relatedness in economic complexity theory [21, 23, 27]. This principle posits that countries are inclined to expand into areas similar to their existing technological and industrial capabilities.

Our findings also reveal that scientific research is a crucial foundation for technological progress in AI, enhancing the comparative advantage of a country in related areas. This suggests that technological breakthroughs in AI often originate from foundational scientific research. The science-technology cross-proximity density, which reflects the closeness between a country’s scientific research and the technological fields on which it is focused, shows a positive and statistically significant relationship with the development of new AI technologies, pointing toward more efficient technological development and innovation. This finding aligns with recent empirical studies showing that scientific capabilities can significantly influence the likelihood of countries developing technologies related to their scientific fields [4, 71].

However, we note an intriguing pattern: the relationship between cross-proximity density and AI specialization has an inverted U-shape. This extends the optimal cognitive proximity theory to the multidimensional space of science and technology, suggesting that knowledge transfer may be impeded if the cognitive proximity between two entities is either too low or too high [53]. This highlights the importance of maintaining a balanced cognitive proximity to foster innovation.

This study also identifies the influence of complex technologies on AI specialization. Although complex technologies positively influence AI specialization, their impact is less pronounced than that of scientific knowledge. This suggests that in rapidly advancing fields, such as AI, incorporating new scientific knowledge into related industries may be more advantageous than simply advancing existing technologies to outpace competitors. These findings challenge the traditional view that increasing technological complexity is the primary pathway to gain a competitive advantage in AI [22]. However, increasing technological complexity appears to be more effective for maintaining existing technological competitiveness than for acquiring new scientific knowledge.

The insight provided by these findings offers valuable guidance for policymakers, particularly in less-developed nations. The results of this study highlight that these countries might lack the infrastructure to foster AI specialization through technical complexity. However, in a rapidly evolving technological landscape, integrating AI into relevant sectors using novel scientific knowledge, even if it is less technically complex, may be more beneficial than emulating the approaches of more advanced nations. This indicates a strategic pivot from prioritizing technological complexity to adopting a balanced strategy that capitalizes on new scientific discoveries.

Our study has several limitations. The publication records used in this study do not fully represent all of the activities in the field of AI, particularly in the humanities, social sciences, and arts, owing to our reliance on data from SCI journals. Nonetheless, by using paper and patent data that predominantly represent academic and inventive activities in AI’s computer science domain, this study contributes to connecting these two dimensions.

Looking ahead, we anticipate that future research could more comprehensively explore how technological breakthroughs in AI, such as those in recent large language models such as ChatGPT, impact national technological competitiveness. This can be achieved using the proposed multidimensional space model.

In conclusion, while enhanced scientific and technological knowledge acts as a catalyst for AI specialization in individual nations, the contemporary landscape presents a more complex picture. Merely expanding knowledge does not guarantee extensive AI specialization; its success depends on relevant application. The interaction between scientific research and technological innovation is crucial for the emergence of new technologies, especially in settings characterized by optimal cognitive proximity. Moreover, the transition from scientific knowledge to technological specialization involves time lags that are steadily shortening in rapidly evolving areas such as AI. For countries seeking technology-related comparative advantages, integrating the latest scientific discoveries into related sectors is more effective than merely increasing the complexity of existing technologies to surpass competitors. This insight is particularly valuable for policymakers in less technologically and economically developed countries, indicating that even with limited technological resources, strategically incorporating relevant AI technologies into closely aligned fields can provide a comparative advantage.

## Supporting information

S1 File (DOCX)
