# Supplementary material for: AI technology specialization and national competitiveness
Source: PLoS One. 2024 Apr 4;19(4):e0301091. doi: 10.1371/journal.pone.0301091 (PMC10994314; doi:10.1371/journal.pone.0301091)
Supplement: S1 File — (DOCX) [file pone.0301091.s001.docx]

**Supporting Information**

**S1 Table. List of AI patent keywords, CPCs, and article key phrases**

| List of AI patent keywords^†^ | |
| --- | --- |
| artificial intelligen, computational, intelligen, neural network, neural_network, bayes network, bayesian network, bayesian-network, bayesian_network, chatbot, learning, learning model, learning algorithm, learning sys, intelligen, classification model, optimiz, training data, training method, convolutional network, anomaly detection, feature ext, prediction model, data mining, decision model, deep learning, deep-earning, deep_learning, genetic algorithm, inductive logic, machine learning, machine_learning, machine-learning, natural langurage, image recogni, reinforcement learning, supervised learning, supervised training, supervised-learning | supervised_learning, swarm intelligen, swarm-intelligen, unsupervised learning, unsupervised training, unsupervised-learning, unsupervised_learning, semi-supervised learning, semi-supervised training, semi_supervised_learning, semi-supervised, connections, expert system, fuzzy logic, transfer learning, transfer-learning, transfer_learning, learning algorithm, learning model, support vector machine, random forest, decision tree, gradient tree boosting, xgboost, adaboost, rankboost, logistic regression, stochastic gradient descent, multilayer perceptron, semantic analysis, dirichlet allocation, multi-agent system, hidden markov model, pattern recogni |
| List of AI patent CPCs^†^ | |
| A61B 5/7264, A61B 5/7267, A63F 13/67, B23K 31/006, B25J 9/161, B29C 66/965, B29C 2945/76979, B60G 2600 /1876, B60G 2600/1878, B60G 2600/1879, B60W 30/06, B60W 30/10, B60W 30/14, B62D 15/0285, B64G 2001/247, E21B 2041/0028, F02D 41/1405, F03D 7/046, F05B 2270/707, F05B 2270/709, F05D 2270/709, F16H 2061/0081, F16H 2061/0084, G01N 29/4481, G01N 33/0034, G01N 2201/1296, G01R 31/2846, G01R 31/3651, G01S 7/417, G05B 13/027, G05B 13/0275, G05B 13/028, G05B 13/0285, G05B 13/029, G05B 13/0295, G05B 2219/33002, G05D 1/00, G05D 1/0088, G06F 11/1476, G06F 11/2257, G06F 15/18, G06F 17/2282, G06F 17/27, G06F 17/28, G06F 17/30029, G06F 17/30247, G06F 17/30401, G06F 17/3043, G06F 17/30522, G06F 17/30654, G06F 17/30663, G06F 17/30666, G06F 17/30669, G06F 17/30672 G06F 17/30684, G06F 17/30687, | G06F 17/3069, G06F 17/30702, G06F 17/30705, G06F 17/30731, G06F 17/30743, G06F 17/30784, G06F 19/24, G06F 19/707, G06F 2207/4824, G06K 7/1482, G06K 9/0, G06N 3/0, G06N 3/004, G06N 7/005, G06N 7/006, G06N 7/046, G06N 99/005, G06T 3/404, G06T 9/00, G06T 2207/2008, G06T 2207/20084, G06T 2207/30236, G06T 2207/30248, G08B 29/186, G10H 2250/151, G10H 2250/311, G10K 2210/3024, G10K 2210/3038, G10L 15, G10L 17, G10L 25/30, G11B 20/10518, H01J 2237/30427, H01M 8/04992, H02H 1/0092, H02P 21/0014, H02P 23/0018, H03H 2017/0208, H03H 2222/04, H04L 25/0254, H04L 25/03165, H04L 41/16, H04L 45/08, H04L 2025/03464, H04L 2025/03554, H04N 21/4665, H04Q 2213/054, H04Q 2213/13343, H04Q 2213/343, H04R 25/507, Y10S 128/924, Y10S 128/925, Y10S 706 |
| List of AI article keyphrases^††^ | |
| artificial intelligen* OR computational intelligen* OR neural network* OR neural_network* OR bayes network* OR bayesian network* OR bayesian-network* OR bayesian_network* OR chatbot* OR learning* OR learning model* OR learning algorithm* OR learning sys* OR intelligen* OR classification model* OR optimiz* OR training data* OR training method* OR convolutional network* OR anomaly detection* OR feature ext* OR feature ext* OR prediction model* OR data mining* OR decision model* OR deep learning* OR deep-learning* OR deep_learning* OR genetic algorithm* OR inductive logic* OR machine learning* OR machine_learning* OR machine-learning* OR natural langurage* OR image recogni* OR reinforcement learning* OR supervised learning* OR supervised training* OR supervised-learning* OR supervised_learning* OR swarm intelligen* OR swarm-intelligen* OR unsupervised learning* OR unsupervised training* OR unsupervised-learning* OR unsupervised_learning* OR semi-supervised learning* OR semi-supervised training* OR semi_supervise. | |
| Notes: † Employing a "wildcard search" or "pattern matching search" is applied to retrieve the bibliometric information with patents from the PATSTAT database. In this case, the percentage sign (%) is used. Souce: WIPO [1]  †† A wildcard search, the asterisk symbol (*) is employed to obtain bibliographic information from the WoS Core Collection. | |

**S2 Table. List of variables and definitions**

| Variables | Abbreviation | Description | Data source |
| --- | --- | --- | --- |
| Entry of Technology | ENTRY | Introduction of new technology with RCA in a country | PATSTAT |
| Exit of Technology | EXIT | Termination of existing technology with RCA in a country | PATSTAT |
| Technology Relatedness Density | TECH_DENSITY | Density of technologies related to a specific technology | PATSTAT |
| Technology Complexity | TECH_COMPLEXITY | Average diversity of countries with RCA in a given technology | PATSTAT |
| Scientific and Technological Cross-Density | CROSS_DENSITY | Average proximity of new technology to a country's scientific & technological portfolio | WoS  PATSTAT |
| Population | POP | Total population of a country | World Bank |
| GDP per Capita | GDP_CAPITA | Gross domestic product divided by midyear population | World Bank |
| Technological Knowledge Stock | TECH_STOCK | Number of patent applications produced by a country | PATSTAT |
| Technology Size | TECH_SIZE | Number of patents per Cooperative Patent Classification (CPC) | PATSTAT |

**S3 Fig. Combined marginal effects of TECH_COMPLEXITY and CROSS_DENSITY**
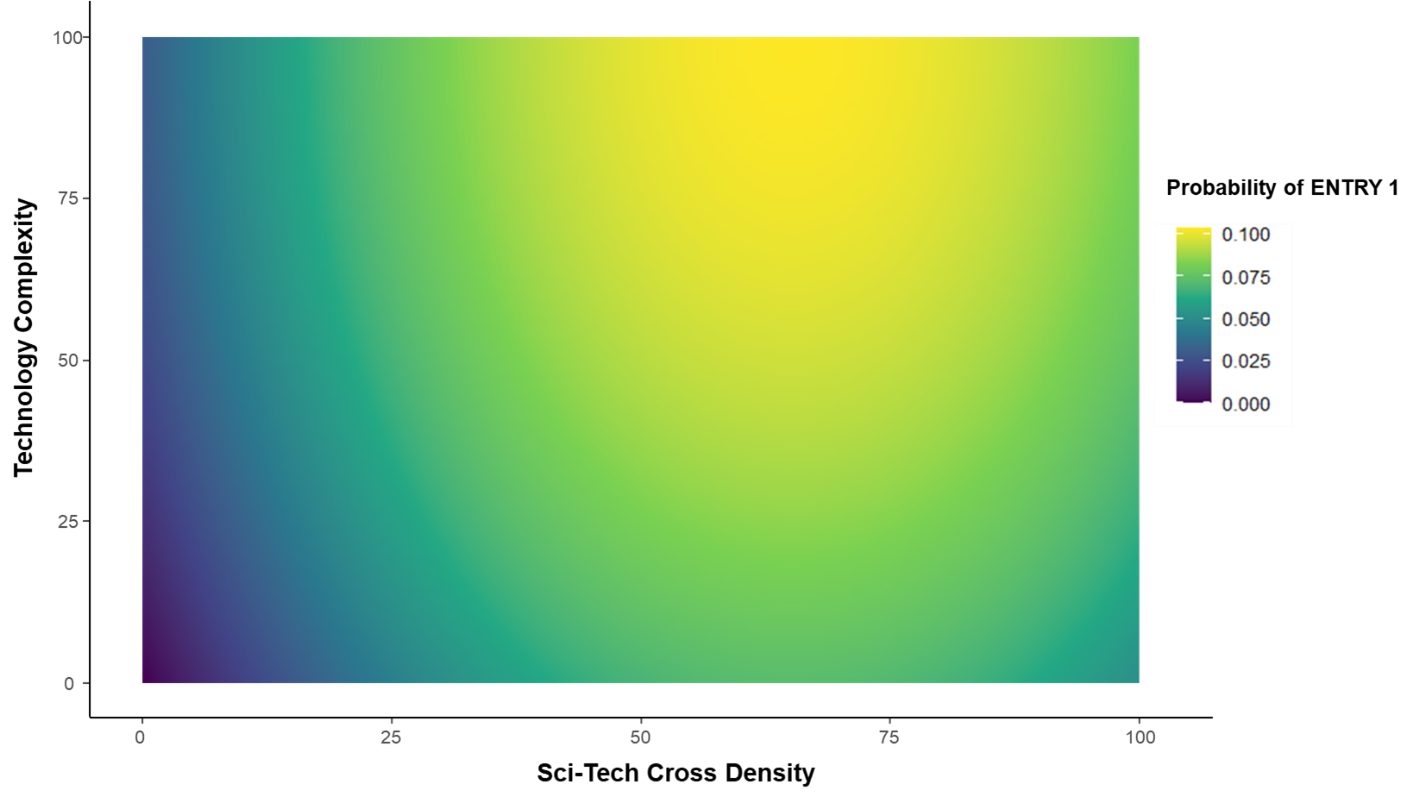


Notes: The brighter yellow shades indicate a stronger combined effect of technology complexity and scientific-technological relatedness density on the probability of ENTRY being 1. Second-degree terms were used to calculate the marginal effects of each variable. All variable coefficients are statistically significant at the 95% confidence level.

**S4 Table. Panel logit regression results (classification schemes)**

| : | Dependent variable: ENTRY | | | EXIT | | |
| --- | --- | --- | --- | --- | --- | --- |
|  | Key phrases  (Title, Abs.) | Key phrases (Title) | CPC symbols | Key phrases  (Title, Abs.) | Key phrases (Title) | CPC symbols |
| TECH_COMPLEXITY | 0.01426***  (0.00160) | 0.01267***  (0.00188) | -0.00683**  (0.00308) | -0.01304***  (0.00321) | -0.01228***  (0.00432) | 0.01612*  (0.00832) |
| TECH_DENSITY | 0.32061***  (0.00472) | 0.26685***  (0.00475) | 0.04666***  (0.00617) | -0.18025***  (0.00685) | -0.16474***  (0.00746) | 0.00545  (0.01404) |
| CROSS_DENSITY | 0.04314***  (0.00220) | 0.03744***  (0.00238) | 0.03276***  (0.00626) | 0.00578  (0.00452) | -0.00044  (0.00554) | -0.00824  (0.01238) |
| TECH_COMPLEXITY_sq | -0.00006**  (0.00004) | -0.00006  (0.00004) | -0.00042***  (0.00007) | 0.00001  (0.00007) | -0.00003  (0.00009) | 0.00040**  (0.00016) |
| TECH_DENSITY_sq | -0.00234***  (0.00006) | -0.00178***  (0.00006) | 0.00027*  (0.00014) | 0.00113***  (0.00008) | 0.00106***  (0.00008) | -0.00104***  (0.00031) |
| CROSS_DENSITY_sq | -0.00042***  (0.00004) | -0.00042***  (0.00005) | -0.00050***  (0.00016) | -0.00002  (0.00007) | 0.00006  (0.00009) | 0.00022  (0.00032) |
| POP | 1.34602***  (0.20128) | 1.29161***  (0.26106) | 0.12742  (0.51051) | 0.51524  (0.62082) | 1.79639**  (0.82830) | -0.60172  (1.42528) |
| GDP_CAPITA | 0.43813***  (0.07199) | 0.17222**  (0.08676) | 0.12439  (0.19999) | 0.32404*  (0.18266) | 0.57281**  (0.22582) | -0.28630  (0.42370) |
| TECH_STOCK | -0.41726***  (0.04072) | -0.19816***  (0.04953) | 0.16544  (0.10354) | 0.56821***  (0.10334) | 0.46714***  (0.13429) | 0.37608  (0.23014) |
| TECH_SIZE | 0.45857***  (0.04184) | 0.46403***  (0.04630) | 0.40700***  (0.08611) | -0.32163***  (0.08680) | -0.48436**8  (0.09844) | -0.0776  (0.19172) |
| Country FEs | YES | YES | YES | YES | YES | YES |
| CPC FEs | YES | YES | YES | YES | YES | YES |
| Period FEs | YES | YES | YES | YES | YES | YES |
| N (group) | 55,154  (11,403) | 41,864  (8,705) | 4,590  (1,080) | 9,691  (3,059) | 6,669  (2,152) | 970  (311) |
| LR ($\chi^{2})$ | 18703.03*** | 13884.53*** | 713.04*** | 2274.06*** | 1608.62*** | 89.79*** |

Notes: Standard errors in parentheses * p<0.1, ** p<0.05, *** p<0.01

The dependent variables ENTRY and EXIT in Model 6 from the main results are analyzed using different AI technology classification schemes. These schemes include key phrases from abstracts and titles of patent documents, key phrases from the titles of patent documents, and the CPC symbols suggested by the World Intellectual Property Organization [1]. https://www.wipo.int/tech_trends/en/artificial_intelligence/patentscope.html

**S5 Table. Panel logit regression results of entry model (Lag** **0–4)**

|  | Dependent variable: ENTRY | | | | |
| --- | --- | --- | --- | --- | --- |
|  | M6 (Lag 0) | M6 (Lag 1) | M6 (Lag 2) | M6 (Lag 3) | M6 (Lag 4) |
| TECH_COMPLEXITY | 0.01579***  (0.00270) | 0.01551***  (0.00159) | 0.01423***  (0.00161) | 0.01426***  (0.00160) | 0.00760***  (0.00161) |
| TECH_DENSITY | 0.55090***  (0.00922) | 0.29944***  (0.00455) | 0.30739***  (0.00463) | 0.32061***  (0.00472) | 0.22319***  (0.00324) |
| CROSS_DENSITY | 0.11203***  (0.00314) | 0.05855***  (0.00208) | 0.05210***  (0.00217) | 0.04314***  (0.00220) | 0.03554***  (0.00216) |
| TECH_COMPLEXITY_sq | -0.00007  (0.00005) | -0.00002  (0.00004) | -0.00003  (0.00004) | -0.00006*  (0.00004) | -0.00007**  (0.00004) |
| TECH_DENSITY_sq | -0.00318***  (0.00016) | -0.00222***  (0.00006) | -0.00225***  (0.00006) | -0.00234***  (0.00006) | -0.00234***  (0.00006) |
| CROSS_DENSITY_sq | -0.00097***  (0.00006) | -0.00033***  (0.00004) | -0.00037***  (0.00004) | -0.00042***  (0.00004) | -0.00055***  (0.00005) |
| POP | 2.11210***  (0.23975) | 1.55846***  (0.19752) | 1.44574***  (0.19888) | 1.34602***  (0.20128) | 1.25800***  (0.20377) |
| GDP_CAPITA | 0.77203***  (0.09342) | 0.53743***  (0.07084) | 0.50835***  (0.07103) | 0.43813***  (0.07199) | 0.39546***  (0.07234) |
| TECH_STOCK | -1.03117***  (0.05196) | -0.36454***  (0.03946) | -0.37079***  (0.03995) | -0.41726***  (0.04072) | -0.26026***  (0.04119) |
| TECH_SIZE | 0.42554***  (0.05766) | 0.46919***  (0.04123) | 0.44697***  (0.04161) | 0.445857***  (0.04184) | 0.46360***  (0.04186) |
| Country FEs | YES | YES | YES | YES | YES |
| CPC FEs | YES | YES | YES | YES | YES |
| Period FEs | YES | YES | YES | YES | YES |
| N (group) | 60,689  (11,898) | 58,746  (11,731) | 56,503  (11,533) | 55,154  (11,403) | 53,229  (11,220) |
| LR ($\chi^{2})$ | 31429.38*** | 20365.80*** | 19325.38*** | 18703.03*** | 16946.67*** |

Notes: Standard errors in parentheses * p<0.1, ** p<0.05, *** p<0.01

The dependent variable ENTRY in Model 6 from the main results is analyzed by varying lagged years. In Model 6 (Lag 0-1), independent and control variables are lagged by zero or one year. In contrast, Model 6 (Lag 2-4) has most variables lagged by one year, while the CROSS_DENSITY is lagged from two to four years.

**S6 Table. Panel logit regression results of exit model (Lag 0–4)**

|  | Dependent variable: EXIT | | | | |
| --- | --- | --- | --- | --- | --- |
|  | M6 (Lag 0) | M6 (Lag 1) | M6 (Lag 2) | M6 (Lag 3) | M6 (Lag 4) |
| TECH_COMPLEXITY | -0.03335***  (0.00999) | -0.01256***  (0.00319) | -0.01307***  (0.00319) | -0.01304***  (0.00321) | -0.01398***  (0.00321) |
| TECH_DENSITY | -0.56658***  (0.03558) | -0.17376***  (0.00698) | -0.17689***  (0.00691) | -0.18025***  (0.00685) | -0.18109***  (0.00683) |
| CROSS_DENSITY | -0.04674***  (0.01218) | -0.01045**  (0.00483) | -0.00123  (0.00464) | 0.00578  (0.00452) | 0.00890**  (0.00445) |
| TECH_COMPLEXITY_sq | -0.00025*  (0.00015) | -0.00001  (0.00007) | -0.00000  (0.00007) | 0.00001  (0.00007) | 0.00000  (0.00007) |
| TECH_DENSITY_sq | -0.00068  (0.00074) | 0.00114***  (0.00008) | 0.00113***  (0.00008) | 0.00113***  (0.0008) | 0.00115***  (0.00008) |
| CROSS_DENSITY_sq | -0.00038  (0.00025) | -0.00000  (0.00008) | -0.00002  (0.00007) | -0.00002  (0.00007) | -0.00008  (0.00007) |
| POP | -4.43734***  (1.19452) | 0.14604  (0.61811) | 0.26026  (0.61913) | 0.51524  (0.62082) | 0.43236  (0.62903) |
| GDP_CAPITA | -0.28694  (0.38664) | 0.28401  (0.18155) | 0.30337*  (0.18177) | 0.32404*  (0.18266) | 0.34537*  (0.18389) |
| TECH_STOCK | 2.04376***  (0.22589) | 0.55428***  (0.10150) | 0.55978***  (0.10269) | 0.56821***  (0.10334) | 0.58182***  (0.10367) |
| TECH_SIZE | -0.28930  (0.19897) | -0.31566***  (0.08701) | -0.31389***  (0.08700) | -0.32163***  (0.08680) | -0.32716***  (0.08701) |
| Country FEs | YES | YES | YES | YES | YES |
| CPC FEs | YES | YES | YES | YES | YES |
| Period FEs | YES | YES | YES | YES | YES |
| N (group) | 9,854  (3,102) | 9,746  (3,076) | 9,746  (3,076) | 9,746  (3,076) | 9,746  (3,076) |
| LR ($\chi^{2})$ | 6301.2-*** | 2286.73*** | 2246.39*** | 2274.06*** | 2287.30*** |

Notes: Standard errors in parentheses * p<0.1, ** p<0.05, *** p<0.01

The dependent variable EXIT in Model 6 from the main results is analyzed by varying lagged years from zero to four years.

**S7 Table. Average marginal effects and net effects.**

|  | Dependent variable: ENTRY | | | | Dependent variable: EXIT | | | |
| --- | --- | --- | --- | --- | --- | --- | --- | --- |
|  | M1 | M2 | M3 | M4 | M1 | M2 | M3 | M4 |
| TECH_DENSITY | 0.00663***  (0.00008) | 0.01045***  (0.00010) | 0.00662***  (0.00001) | 0.01033***  (0.00010) | –0.01196***  (0.00026) | –0.02186***  (0.00060) | –0.01196***  (0.00026) | –0.02196***  (0.00060) |
| TECH_COMPLEXITY | 0.00028***  (0.00004) | 0.00045***  (0.00000) | 0.00061***  (0.00011) | 0.00075***  (0.00011) | –0.00129***  (0.00029) | –0.00162***  (0.00028) | –0.00128  (0.00080) | –0.00167**  (0.0032) |
| CROSS_DENSITY | 0.00093***  (0.00004) | 0.00081***  (0.00004) | 0.00092***  (0.00004) | 0.00173***  (0.00010) | 0.00061***  (0.00023) | 0.00057***  (0.00021) | 0.00061***  (0.00023) | 0.00123**  (0.00073) |
| DENSITY_sq |  | –0.00007***  (0.00000) |  | –0.00006***  (0.00000) |  | 0.00013***  (0.00000) |  | 0.00013***  (0.00001) |
| TECH_COMPLEXITY_sq |  |  | –0.00000***  (0.00000) | –0.00000***  (0.00000) |  |  | –0.00000  (0.00001) | 0.00000  (0.00001) |
| CROSS_DENSITY_sq |  |  |  | –0.00001***  (0.00000) |  |  |  | –0.00001  (0.00001) |
| POP | 0.04913***  (0.00509) | 0.04193***  (0.00517) | 0.04868***  (0.00510) | 0.03949***  (0.00525) | 0.01248  (0.05729) | 0.02379  (0.05517) | 0.01246  (0.05739) | 0.02522  (0.05538) |
| GDP_CAPITA | 0.01615***  (0.00188) | 0.01374***  (0.00517) | 0.01589***  (0.00188) | 0.01206***  (0.00191) | 0.04182**  (0.05729) | 0.04259**  (0.01647) | 0.04181**  (0.01554) | 0.04303**  (0.01645) |
| TECH_STOCK | –0.00434***  (0.00101) | –0.01029***  (0.00517) | –0.0042***  (0.00101) | –0.00110***  (0.00105) | 0.04722***  (0.00823) | 0.06059***  (0.00883) | 0.04722***  (0.00823) | 0.06032***  (0.00881) |
| TECH_SIZE | 0.01409***  (0.00117) | 0.01295***  (0.00115) | 0.01411***  (0.00116) | 0.01206***  (0.00115) | –0.04001***  (0.00781) | –0.03644***  (0.00758) | –0.04000***  (0.00780) | –0.03650***  (0.00759) |
| Net effects^†^ |  | 0.010459 | 0.000614 | 0.001739 |  | -0.021867 |  |  |
| Country FEs | YES | YES | YES | YES | YES | YES | YES | YES |
| CPC FEs | YES | YES | YES | YES | YES | YES | YES | YES |
| Period FEs | YES | YES | YES | YES | YES | YES | YES | YES |
| N (group) | 41,153  (8,284) | 13,496  (3,027) | 16,730  (3,530) | 37,726  (7,794) | 8,673  (2,639) | 1,004  (414) | 5,489  (1,587) | 4,191  (1,471) |
| Log Liklihood | -17322.89 | -16591.83 | -17317.15 | -16524.15 | -4491.973 | -4347.494 | -4491.973 | -4346.948 |

Notes: Standard errors in parentheses * p<0.1, ** p<0.05, *** p<0.01

† The net effects were calculated only for quadratic terms that have statistically significant marginal effects at the 95% level or higher.

**S8 Table. Panel logit regression results (excluding observations of US and CN)**

|  | Dependent variable: ENTRY | | | | | Dependent variable: EXIT | | | | |
| --- | --- | --- | --- | --- | --- | --- | --- | --- | --- | --- |
|  | M1 | M2 | M3 | M4 | M1 | | M2 | M3 | M4 |  |
| TECH_DENSITY | 0.2331***  (0.0035) | 0.3270***  (0.0050) | 0.2331***  (0.0035) | 0.3256***  (0.0050) | –0.11367***  (0.0037) | | –0.1867***  (0.0077) | –0.1138***  (0.0037) | –0.1869***  (0.0078) |  |
| TECH_  COMPLEXITY | 0.0085***  (0.0016) | 0.0134***  (0.0016) | 0.0072***  (0.0017) | 0.0135***  (0.0017) | –0.0109***  (0.0033) | | –0.0132***  (0.0033) | –0.0098***  (0.0034) | –0.0122***  (0.0034) |  |
| CROSS_DENSITY | 0.0329***  (0.0017) | 0.0303***  (0.0017) | 0.0328***  (0.0017) | 0.0447***  (0.0023) | 0.0049**  (0.0025) | | 0.0053**  (0.0025) | 0.0050**  (0.0025) | 0.0057  (0.0051) |  |
| DENSITY_sq |  | –0.0023***  (0.0001) |  | –0.0022***  (0.0001) |  | | 0.0011***  (0.0001) |  | 0.0011***  (0.0001) |  |
| TECH_  COMPLEXITY_sq |  |  | –0.0001**  (0.0001) | –0.0001**  (0.0001) |  | |  | 0.0000  (0.0001) | 0.0000  (0.0001) |  |
| CROSS_DENSITY_sq |  |  |  | –0.0004***  (0.0001) |  | |  |  | –0.0000  (0.0001) |  |
| POP | 1.5686***  (0.2015) | 1.3172***  (0.2056) | 1.5546***  (0.2017) | 1.1690***  (0.2086) | 0.9308  (0.6504) | | 1.1251*  (0.6572) | 0.9609  (0.6504) | 1.1597*  (0.6579) |  |
| GDP_CAPITA | 0.8463***  (0.0754) | 0.7229***  (0.07621) | 0.8334***  (0.0755) | 0.6072***  (0.0777) | 0.5675***  (0.2032) | | 0.6279***  (0.2105) | 0.5772***  (0.2032) | 0.6367***  (0.2106) |  |
| TECH_STOCK | –0.0867***  (0.0373) | –0.2723***  (0.0385) | –0.0837***  (0.0373) | –0.3079***  (0.0388) | 0.4002***  (0.0911) | | 0.5127***  (0.0954) | 0.3992***  (0.0910) | 0.5107***  (0.0955) |  |
| TECH_SIZE | 0.4706***  (0.0424) | 0.4480***  (0.0426) | 0.4701***  (0.0423) | 0.4403***  (0.0427) | –0.3771***  (0.0919) | | –0.3686***  (0.0932) | –0.3788***  (0.0919) | –0.3711***  (0.0931) |  |
| Country FEs | YES | YES | YES | YES | YES | | YES | YES | YES |  |
| CPC FEs | YES | YES | YES | YES | YES | | YES | YES | YES |  |
| Period FEs | YES | YES | YES | YES | YES | | YES | YES | YES |  |
| N (group) | 53,107  (10,655) | 53,107  (10,655) | 53,107  (10,655) | 53,107  (10,655) | 8,419  (2,691) | | 8,419  (2,691) | 8,419  (2,691) | 8,419  (2,691) |  |
| LR (χ^2) | 17426*** | 17435*** | 17620*** | 18121*** | 1747*** | | 1864*** | 1748*** | 1865*** |  |

Notes: Standard errors are shown in parentheses. Significance levels are indicated as follows: * p < 0.1, ** p < 0.05, *** p < 0.01. 'US' and 'CN' denote the United States and China, respectively. The dependent variables, 'ENTRY' or 'EXIT', are binary (0 or 1). The independent and control variables are lagged by one year, except for 'CROSS_DENSITY', which is lagged by three years. The term 'sq' denotes a squared term.

**Reference**

[1] WIPO, 2019, *“PATENTSCOPE artificial intelligence index”*. Available at: https://www.wipo.int/tech_trends/en/artificial_intelligence/patentscope.html.
